# Supplementary material for: Exploring perception and attitude of nursing students towards interprofessional education in Saudi Arabia
Source: PLoS One. 2024 Oct 4;19(10):e0311570. doi: 10.1371/journal.pone.0311570 (PMC11452034; doi:10.1371/journal.pone.0311570)
Supplement: S1 Raw data — (PDF) [file pone.0311570.s001.pdf]

Research raw data

| Serial no. | Age (Years) | Academic year | V1- Learning with other |
|---|---|---|---|
| 1 | 19 - 21 | 3rd | Strongly Disagree |
| 2 | 19 - 21 | 3rd | Agree |
| 3 | 19 - 21 | 3rd | Neutral |
| 4 | 19 - 21 | 3rd | Neutral |
| 5 | 19 - 21 | 3rd | Agree |
| 6 | 19 - 21 | 3rd | Strongly agree |
| 7 | 19 - 21 | 3rd | Strongly agree |
| 8 | 19 - 21 | 3rd | Neutral |
| 9 | 19 - 21 | 3rd | Strongly Disagree |
| 10 | 19 - 21 | 3rd | Strongly agree |
| 11 | 19 - 21 | 3rd | Agree |
| 12 | 19 - 21 | 3rd | Agree |
| 13 | 19 - 21 | 3rd | Neutral |
| 14 | 19 - 21 | 3rd | Strongly Disagree |
| 15 | 19 - 21 | 3rd | Disagree |
| 16 | 19 - 21 | 3rd | Neutral |
| 17 | 19 - 21 | 3rd | Disagree |
| 18 | 19 - 21 | 3rd | Strongly agree |
| 19 | 19 - 21 | 3rd | Agree |
| 20 | 19 - 21 | 3rd | Strongly agree |
| 21 | 19 - 21 | 3rd | Strongly Disagree |
| 22 | 19 - 21 | 3rd | Neutral |
| 23 | 19 - 21 | 3rd | Agree |
| 24 | 19 - 21 | 3rd | Neutral |
| 25 | 19 - 21 | 3rd | Neutral |
| 26 | 19 - 21 | 3rd | Strongly agree |
| 27 | 19 - 21 | 3rd | Strongly agree |
| 28 | 19 - 21 | 3rd | Agree |
| 29 | 19 - 21 | 3rd | Strongly Disagree |
| 30 | 19 - 21 | 3rd | Disagree |
| 31 | 19 - 21 | 3rd | Agree |
| 32 | 19 - 21 | 3rd | Strongly agree |
| 33 | 19 - 21 | 3rd | Agree |
| 34 | 19 - 21 | 3rd | Strongly agree |
| 35 | 19 - 21 | 3rd | Strongly agree |
| 36 | 19 - 21 | 3rd | Strongly agree |
| 37 | 19 - 21 | 3rd | Agree |
| 38 | 19 - 21 | 3rd | Strongly agree |
| 39 | 19 - 21 | 3rd | Strongly agree |
| 40 | 19 - 21 | 3rd | Neutral |
| 41 | 19 - 21 | 3rd | Strongly agree |
| 42 | 19 - 21 | 3rd | Strongly Disagree |
| 43 | 19 - 21 | 3rd | Neutral |
| 44 | 19 - 21 | 3rd | Strongly agree |
| 45 | 19 - 21 | 3rd | Agree |
| 46 | 19 - 21 | 3rd | Neutral |
| 47 | 19 - 21 | 3rd | Agree |
| 48 | 19 - 21 | 3rd | Strongly agree |
| 49 | 19 - 21 | 3rd | Strongly agree |
| 50 | 19 - 21 | 3rd | Disagree |
| 51 | 19 - 21 | 3rd | Strongly agree |
| 52 | 19 - 21 | 3rd | Neutral |

| | | | |
|---|---|---|---|
| 53 | 19 - 21 | 3rd | Strongly agree |
| 54 | 19 - 21 | 3rd | Strongly agree |
| 55 | 19 - 21 | 3rd | Neutral |
| 56 | 19 - 21 | 3rd | Strongly Disagree |
| 57 | 19 - 21 | 3rd | Agree |
| 58 | 19 - 21 | 3rd | Neutral |
| 59 | 19 - 21 | 3rd | Neutral |
| 60 | 19 - 21 | 3rd | Neutral |
| 61 | 19 - 21 | 3rd | Strongly agree |
| 62 | 19 - 21 | 3rd | Strongly agree |
| 63 | 19 - 21 | 3rd | Strongly Disagree |
| 64 | 19 - 21 | 3rd | Agree |
| 65 | 19 - 21 | 3rd | Agree |
| 66 | 19 - 21 | 3rd | Disagree |
| 67 | 19 - 21 | 3rd | Agree |
| 68 | 19 - 21 | 3rd | Strongly agree |
| 69 | 19 - 21 | 3rd | Agree |
| 70 | 19 - 21 | 3rd | Agree |
| 71 | 19 - 21 | 3rd | Agree |
| 72 | 19 - 21 | 3rd | Strongly agree |
| 73 | 19 - 21 | 3rd | Strongly agree |
| 74 | 19 - 21 | 3rd | Agree |
| 75 | 19 - 21 | 3rd | Agree |
| 76 | 19 - 21 | 3rd | Agree |
| 77 | 19 - 21 | 3rd | Neutral |
| 78 | 19 - 21 | 3rd | Strongly agree |
| 79 | 19 - 21 | 3rd | Neutral |
| 80 | 19 - 21 | 3rd | Strongly agree |
| 81 | 19 - 21 | 3rd | Strongly agree |
| 82 | 19 - 21 | 3rd | Strongly agree |
| 83 | 19 - 21 | 3rd | Agree |
| 84 | 19 - 21 | 3rd | Strongly agree |
| 85 | 19 - 21 | 3rd | Agree |
| 86 | 19 - 21 | 3rd | Agree |
| 87 | 19 - 21 | 3rd | Neutral |
| 88 | 19 - 21 | 3rd | Neutral |
| 89 | 19 - 21 | 3rd | Agree |
| 90 | 19 - 21 | 3rd | Neutral |
| 91 | 19 - 21 | 3rd | Neutral |
| 92 | < 19 | 3rd | Strongly Disagree |
| 93 | 19 - 21 | 3rd | Strongly agree |
| 94 | 19 - 21 | 3rd | Agree |
| 95 | 19 - 21 | 3rd | Agree |
| 96 | 19 - 21 | 3rd | Strongly agree |
| 97 | 19 - 21 | 3rd | Neutral |
| 98 | 19 - 21 | 3rd | Strongly agree |
| 99 | 19 - 21 | 3rd | Agree |
| 100 | 19 - 21 | 3rd | Agree |
| 101 | 19 - 21 | 3rd | Strongly agree |
| 102 | 19 - 21 | 3rd | Agree |
| 103 | 19 - 21 | 3rd | Agree |
| 104 | 19 - 21 | 3rd | Agree |
| 105 | 19 - 21 | 3rd | Agree |
| 106 | 19 - 21 | 3rd | Strongly agree |
| 107 | 19 - 21 | 3rd | Neutral |
| 108 | 19 - 21 | 3rd | Neutral |
| 109 | > 21 | 3rd | Agree |
| 110 | 19 - 21 | 3rd | Neutral |
| 111 | 19 - 21 | 3rd | Strongly agree |

| | | | |
|---|---|---|---|
| 112 | 19 - 21 | 3rd | Agree |
| 113 | 19 - 21 | 3rd | Strongly agree |
| 114 | 19 - 21 | 3rd | Agree |
| 115 | 19 - 21 | 3rd | Agree |
| 116 | 19 - 21 | 3rd | Strongly agree |
| 117 | 19 - 21 | 3rd | Neutral |
| 118 | 19 - 21 | 3rd | Strongly agree |
| 119 | 19 - 21 | 3rd | Neutral |
| 120 | 19 - 21 | 3rd | Strongly Disagree |
| 121 | 19 - 21 | 3rd | Strongly agree |
| 122 | 19 - 21 | 3rd | Agree |
| 123 | 19 - 21 | 3rd | Strongly agree |
| 124 | 19 - 21 | 3rd | Neutral |
| 125 | 19 - 21 | 3rd | Strongly agree |
| 126 | 19 - 21 | 3rd | Strongly agree |
| 127 | 19 - 21 | 3rd | Agree |
| 128 | > 21 | 3rd | Neutral |
| 129 | 19 - 21 | 3rd | Strongly agree |
| 130 | 19 - 21 | 3rd | Agree |
| 131 | 19 - 21 | 3rd | Strongly agree |
| 132 | 19 - 21 | 3rd | Strongly agree |
| 133 | 19 - 21 | 3rd | Strongly agree |
| 134 | 19 - 21 | 3rd | Strongly agree |
| 135 | 19 - 21 | 3rd | Strongly agree |
| 136 | 19 - 21 | 3rd | Strongly agree |
| 137 | 19 - 21 | 3rd | Strongly agree |
| 138 | 19 - 21 | 3rd | Agree |
| 139 | 19 - 21 | 3rd | Agree |
| 140 | 19 - 21 | 3rd | Strongly agree |
| 141 | 19 - 21 | 3rd | Strongly agree |
| 142 | 19 - 21 | 3rd | Strongly agree |
| 143 | 19 - 21 | 3rd | Neutral |
| 144 | 19 - 21 | 3rd | Strongly agree |
| 145 | 19 - 21 | 3rd | Strongly agree |
| 146 | 19 - 21 | 3rd | Agree |
| 147 | 19 - 21 | 3rd | Disagree |
| 148 | 19 - 21 | 3rd | Strongly agree |
| 149 | 19 - 21 | 3rd | Strongly agree |
| 150 | 19 - 21 | 3rd | Strongly Disagree |
| 151 | 19 - 21 | 3rd | Neutral |
| 152 | 19 - 21 | 3rd | Strongly agree |
| 153 | > 21 | 3rd | Disagree |
| 154 | 19 - 21 | 3rd | Neutral |
| 158 | > 21 | 3rd | Strongly Disagree |
| 159 | > 21 | 4th | Strongly agree |
| 160 | 19 - 21 | 4th | Agree |
| 161 | 19 - 21 | 3rd | Disagree |
| 168 | 19 - 21 | 3rd | Strongly Disagree |
| 169 | 19 - 21 | 4th | Agree |
| 170 | 19 - 21 | 4th | Strongly agree |
| 181 | 19 - 21 | 4th | Strongly agree |
| 182 | 19 - 21 | 4th | Agree |
| 189 | 19 - 21 | 4th | Strongly agree |
| 155 | 19 - 21 | 4th | Agree |
| 156 | 19 - 21 | 4th | Neutral |
| 157 | 19 - 21 | 4th | Agree |
| 162 | > 21 | 4th | Strongly agree |
| 163 | > 21 | 4th | Strongly agree |
| 164 | > 21 | 4th | Strongly agree |

| | | | |
|---|---|---|---|
| 165 | > 21 | 4th | Strongly agree |
| 166 | 19 - 21 | 4th | Strongly Disagree |
| 167 | 19 - 21 | 4th | Agree |
| 171 | 19 - 21 | 4th | Strongly agree |
| 172 | > 21 | 4th | Agree |
| 173 | > 21 | 4th | Neutral |
| 174 | > 21 | 4th | Strongly agree |
| 175 | 19 - 21 | 4th | Agree |
| 176 | 19 - 21 | 4th | Strongly agree |
| 177 | 19 - 21 | 4th | Strongly agree |
| 178 | > 21 | 4th | Strongly Disagree |
| 179 | > 21 | 4th | Strongly agree |
| 180 | > 21 | 4th | Disagree |
| 183 | 19 - 21 | 4th | Agree |
| 184 | 19 - 21 | 4th | Strongly agree |
| 185 | 19 - 21 | 4th | Agree |
| 186 | > 21 | 4th | Agree |
| 187 | > 21 | 4th | Strongly agree |
| 188 | 19 - 21 | 4th | Neutral |
| 190 | > 21 | 4th | Neutral |
| 191 | 19 - 21 | 4th | Strongly agree |
| 192 | > 21 | 4th | Agree |
| 193 | > 21 | 4th | Strongly agree |
| 194 | 19 - 21 | 4th | Strongly agree |
| 195 | 19 - 21 | 4th | Neutral |
| 196 | 19 - 21 | 4th | Strongly agree |
| 197 | 19 - 21 | 4th | Agree |
| 198 | > 21 | 4th | Strongly agree |
| 199 | > 21 | 4th | Strongly agree |
| 200 | 19 - 21 | 4th | Strongly agree |
| 201 | 19 - 21 | 4th | Neutral |
| 202 | > 21 | 4th | Strongly agree |
| 203 | > 21 | 4th | Agree |
| 204 | > 21 | 4th | Strongly agree |
| 205 | > 21 | 4th | Strongly agree |
| 206 | 19 - 21 | 4th | Strongly agree |
| 207 | > 21 | 4th | Strongly agree |
| 208 | 19 - 21 | 4th | Strongly agree |
| 209 | 19 - 21 | 4th | Strongly agree |
| 210 | > 21 | 4th | Agree |
| 211 | > 21 | 4th | Strongly Disagree |
| 212 | > 21 | 4th | Agree |
| 213 | 19 - 21 | 4th | Strongly agree |
| 214 | 19 - 21 | 4th | Strongly agree |
| 215 | 19 - 21 | 4th | Agree |
| 216 | > 21 | 4th | Strongly agree |
| 217 | 19 - 21 | 4th | Strongly agree |
| 218 | 19 - 21 | 4th | Agree |
| 219 | 19 - 21 | 4th | Strongly agree |
| 220 | 19 - 21 | 4th | Strongly agree |
| 221 | > 21 | 4th | Strongly agree |
| 222 | 19 - 21 | 4th | Agree |
| 223 | 19 - 21 | 4th | Strongly agree |
| 224 | 19 - 21 | 4th | Strongly agree |
| 225 | 19 - 21 | 4th | Agree |
| 226 | 19 - 21 | 4th | Strongly agree |
| 227 | 19 - 21 | 4th | Strongly agree |
| 228 | 19 - 21 | 4th | Disagree |
| 229 | 19 - 21 | 4th | Strongly agree |

| | | | |
|---|---|---|---|
| 230 | 19 - 21 | 4th | Strongly agree |
| 231 | 19 - 21 | 4th | Strongly agree |
| 232 | 19 - 21 | 4th | Strongly agree |
| 233 | 19 - 21 | 4th | Strongly agree |
| 234 | 19 - 21 | 4th | Strongly agree |
| 235 | 19 - 21 | 4th | Agree |
| 236 | 19 - 21 | 4th | Agree |
| 237 | 19 - 21 | 4th | Strongly agree |
| 238 | 19 - 21 | 4th | Agree |
| 239 | 19 - 21 | 4th | Strongly agree |
| 240 | 19 - 21 | 4th | Agree |
| 241 | 19 - 21 | 4th | Strongly agree |
| 242 | 19 - 21 | 4th | Strongly agree |
| 243 | 19 - 21 | 4th | Strongly agree |
| 244 | 19 - 21 | 4th | Agree |
| 245 | 19 - 21 | 4th | Strongly agree |
| 246 | 19 - 21 | 4th | Neutral |
| 247 | 19 - 21 | 4th | Agree |
| 248 | 19 - 21 | 4th | Agree |
| 249 | 19 - 21 | 4th | Strongly agree |
| 250 | 19 - 21 | 4th | Strongly agree |
| 251 | 19 - 21 | 4th | Strongly agree |
| 252 | 19 - 21 | 4th | Neutral |
| 253 | 19 - 21 | 4th | Strongly agree |
| 254 | 19 - 21 | 4th | Strongly agree |
| 255 | 19 - 21 | 4th | Strongly agree |
| 256 | 19 - 21 | 4th | Strongly agree |
| 257 | 19 - 21 | 4th | Strongly agree |
| 258 | 19 - 21 | 4th | Agree |
| 259 | 19 - 21 | 4th | Neutral |
| 260 | 19 - 21 | 4th | Strongly agree |
| 261 | 19 - 21 | 4th | Neutral |
| 262 | 19 - 21 | 4th | Strongly agree |
| 263 | 19 - 21 | 4th | Agree |
| 264 | 19 - 21 | 4th | Strongly agree |
| 265 | 19 - 21 | 4th | Neutral |
| 266 | 19 - 21 | 4th | Neutral |
| 267 | 19 - 21 | 4th | Agree |
| 268 | 19 - 21 | 4th | Strongly agree |
| 269 | 19 - 21 | 4th | Agree |
| 270 | 19 - 21 | 4th | Strongly agree |
| 271 | 19 - 21 | 4th | Neutral |
| 272 | 19 - 21 | 4th | Agree |
| 273 | 19 - 21 | 4th | Strongly agree |
| 274 | 19 - 21 | 4th | Neutral |
| 275 | 19 - 21 | 4th | Strongly agree |
| 276 | 19 - 21 | 4th | Strongly Disagree |
| 277 | 19 - 21 | 4th | Agree |
| 278 | 19 - 21 | 4th | Strongly agree |
| 279 | 19 - 21 | 4th | Agree |
| 280 | 19 - 21 | 4th | Strongly agree |
| 281 | 19 - 21 | 4th | Agree |
| 282 | 19 - 21 | 4th | Neutral |
| 283 | 19 - 21 | 4th | Agree |
| 284 | 19 - 21 | 4th | Strongly agree |
| 285 | 19 - 21 | 4th | Strongly agree |
| 286 | 19 - 21 | 4th | Agree |
| 287 | 19 - 21 | 4th | Strongly agree |
| 288 | 19 - 21 | 4th | Agree |

| | | | |
|---|---|---|---|
| 289 | > 21 | 4th | Neutral |
| 290 | > 21 | 4th | Strongly agree |
| 291 | > 21 | 4th | Agree |
| 292 | > 21 | 4th | Strongly agree |
| 293 | > 21 | 4th | Agree |
| 294 | 19 - 21 | 4th | Strongly agree |
| 295 | 19 - 21 | 4th | Neutral |
| 296 | 19 - 21 | 4th | Strongly agree |
| 297 | 19 - 21 | 4th | Agree |
| 298 | 19 - 21 | 4th | Strongly agree |
| 299 | 19 - 21 | 4th | Agree |
| 300 | > 21 | 4th | Agree |
| 301 | 19 - 21 | 4th | Agree |
| 302 | > 21 | 4th | Agree |
| 303 | > 21 | 4th | Agree |
| 304 | 19 - 21 | 4th | Agree |
| 305 | 19 - 21 | 4th | Strongly agree |
| 306 | > 21 | 4th | Agree |
| 307 | > 21 | 4th | Neutral |
| 308 | > 21 | 4th | Strongly agree |
| 309 | 19 - 21 | 4th | Agree |
| 310 | > 21 | 4th | Strongly agree |
| 311 | > 21 | 4th | Strongly agree |

| V2- Patients would ultir | V3- Shared learning w | V4- Learning with healt | V5- Communication sk |
|---|---|---|---|
| Strongly agree | Strongly agree | Strongly agree | Agree |
| Strongly agree | Strongly agree | Neutral | Agree |
| Neutral | Neutral | Neutral | Neutral |
| Disagree | Neutral | Disagree | Strongly agree |
| Strongly agree | Strongly agree | Strongly agree | Neutral |
| Agree | Strongly agree | Strongly agree | Strongly agree |
| Neutral | Strongly agree | Neutral | Strongly agree |
| Neutral | Neutral | Neutral | Neutral |
| Strongly disagree | Strongly disagree | Strongly disagree | Agree |
| Strongly agree | Strongly agree | Agree | Agree |
| Disagree | Disagree | Agree | Strongly agree |
| Strongly agree | Agree | Agree | Neutral |
| Neutral | Strongly agree | Strongly agree | Strongly agree |
| Strongly disagree | Strongly disagree | Strongly disagree | Strongly disagree |
| Neutral | Agree | Disagree | Disagree |
| Strongly agree | Strongly agree | Agree | Agree |
| Strongly disagree | Agree | Agree | Agree |
| Strongly agree | Strongly agree | Strongly agree | Strongly agree |
| Agree | Agree | Agree | Agree |
| Strongly agree | Agree | Agree | Agree |
| Strongly disagree | Strongly disagree | Strongly disagree | Strongly disagree |
| Neutral | Agree | Agree | Agree |
| Agree | Strongly agree | Agree | Neutral |
| Strongly agree | Strongly agree | Strongly agree | Strongly agree |
| Neutral | Agree | Agree | Strongly agree |
| Agree | Strongly agree | Strongly agree | Strongly agree |
| Agree | Strongly agree | Agree | Neutral |
| Disagree | Strongly agree | Agree | Neutral |
| Strongly disagree | Strongly disagree | Strongly disagree | Strongly disagree |
| Strongly disagree | Agree | Disagree | Agree |
| Strongly agree | Strongly agree | Strongly agree | Neutral |
| Strongly agree | Strongly agree | Agree | Strongly agree |
| Agree | Agree | Agree | Agree |
| Strongly agree | Agree | Strongly agree | Agree |
| Strongly agree | Strongly agree | Agree | Strongly agree |
| Strongly agree | Strongly agree | Strongly agree | Strongly agree |
| Agree | Agree | Agree | Agree |
| Strongly agree | Strongly agree | Strongly agree | Strongly agree |
| Strongly agree | Strongly agree | Strongly agree | Strongly agree |
| Agree | Strongly agree | Neutral | Strongly agree |
| Agree | Strongly agree | Strongly agree | Agree |
| Strongly agree | Agree | Agree | Agree |
| Agree | Neutral | Disagree | Agree |
| Strongly agree | Strongly agree | Strongly agree | Strongly agree |
| Neutral | Neutral | Agree | Agree |
| Neutral | Neutral | Neutral | Neutral |
| Agree | Agree | Agree | Agree |
| Strongly agree | Agree | Strongly agree | Neutral |
| Neutral | Strongly agree | Strongly agree | Strongly agree |
| Neutral | Agree | Agree | Agree |
| Agree | Agree | Agree | Strongly agree |
| Agree | Strongly agree | Agree | Neutral |

| | | | |
|---|---|---|---|
| Strongly agree | Strongly agree | Agree | Agree |
| Strongly agree | Strongly agree | Strongly agree | Agree |
| Agree | Agree | Agree | Agree |
| Disagree | Strongly disagree | Strongly disagree | Disagree |
| Agree | Agree | Agree | Agree |
| Neutral | Agree | Neutral | Neutral |
| Neutral | Neutral | Neutral | Agree |
| Neutral | Neutral | Neutral | Neutral |
| Strongly agree | Strongly agree | Neutral | Strongly agree |
| Strongly agree | Strongly agree | Neutral | Strongly agree |
| Disagree | Disagree | Disagree | Disagree |
| Strongly disagree | Agree | Agree | Agree |
| Strongly agree | Agree | Agree | Agree |
| Neutral | Agree | Disagree | Disagree |
| Agree | Agree | Agree | Neutral |
| Strongly agree | Strongly agree | Strongly agree | Strongly agree |
| Agree | Agree | Agree | Agree |
| Neutral | Strongly agree | Agree | Neutral |
| Strongly agree | Strongly agree | Agree | Agree |
| Neutral | Strongly agree | Neutral | Neutral |
| Agree | Agree | Strongly agree | Agree |
| Agree | Neutral | Strongly agree | Strongly agree |
| Agree | Agree | Agree | Agree |
| Neutral | Agree | Strongly agree | Agree |
| Agree | Neutral | Agree | Neutral |
| Agree | Strongly agree | Neutral | Strongly agree |
| Agree | Agree | Agree | Agree |
| Strongly agree | Strongly agree | Strongly agree | Agree |
| Strongly agree | Agree | Neutral | Agree |
| Neutral | Strongly disagree | Disagree | Strongly agree |
| Agree | Neutral | Agree | Neutral |
| Strongly agree | Strongly agree | Strongly agree | Strongly agree |
| Agree | Agree | Neutral | Agree |
| Agree | Agree | Agree | Agree |
| Agree | Agree | Agree | Agree |
| Neutral | Disagree | Neutral | Strongly disagree |
| Agree | Neutral | Agree | Agree |
| Neutral | Disagree | Neutral | Neutral |
| Agree | Strongly agree | Agree | Agree |
| Agree | Agree | Agree | Agree |
| Agree | Agree | Agree | Agree |
| Strongly agree | Agree | Agree | Neutral |
| Agree | Agree | Agree | Agree |
| Strongly agree | Strongly agree | Strongly agree | Strongly agree |
| Agree | Agree | Agree | Agree |
| Strongly agree | Strongly agree | Strongly agree | Strongly agree |
| Strongly agree | Strongly agree | Neutral | Agree |
| Strongly agree | Agree | Agree | Agree |
| Strongly agree | Agree | Agree | Strongly agree |
| Agree | Agree | Agree | Agree |
| Agree | Strongly agree | Neutral | Strongly agree |
| Neutral | Strongly agree | Agree | Agree |
| Agree | Agree | Agree | Agree |
| Strongly agree | Strongly agree | Strongly agree | Strongly agree |
| Agree | Agree | Agree | Agree |
| Strongly agree | Strongly agree | Strongly agree | Strongly agree |
| Agree | Agree | Agree | Agree |
| Neutral | Neutral | Neutral | Neutral |
| Neutral | Strongly agree | Agree | Strongly agree |

| | | | |
|---|---|---|---|
| Agree | Agree | Agree | Agree |
| Strongly agree | Strongly agree | Strongly agree | Strongly agree |
| Agree | Agree | Agree | Neutral |
| Agree | Agree | Agree | Agree |
| Strongly agree | Strongly agree | Strongly agree | Strongly agree |
| Agree | Neutral | Agree | Neutral |
| Strongly agree | Strongly agree | Strongly agree | Strongly agree |
| Neutral | Neutral | Neutral | Neutral |
| Strongly disagree | Strongly disagree | Strongly disagree | Strongly disagree |
| Agree | Strongly agree | Agree | Agree |
| Agree | Agree | Agree | Agree |
| Strongly agree | Agree | Agree | Agree |
| Neutral | Neutral | Neutral | Disagree |
| Strongly agree | Strongly agree | Strongly agree | Strongly agree |
| Strongly agree | Strongly agree | Strongly agree | Strongly agree |
| Agree | Agree | Agree | Neutral |
| Agree | Agree | Agree | Agree |
| Strongly agree | Strongly agree | Strongly agree | Strongly agree |
| Agree | Agree | Agree | Agree |
| Agree | Strongly agree | Strongly agree | Strongly agree |
| Strongly agree | Strongly agree | Strongly agree | Strongly agree |
| Agree | Strongly agree | Agree | Agree |
| Agree | Strongly agree | Strongly agree | Agree |
| Agree | Agree | Neutral | Disagree |
| Strongly agree | Strongly agree | Strongly agree | Strongly agree |
| Agree | Agree | Strongly agree | Agree |
| Strongly agree | Strongly agree | Strongly agree | Strongly agree |
| Strongly agree | Strongly agree | Agree | Strongly agree |
| Strongly agree | Strongly agree | Strongly agree | Strongly agree |
| Strongly agree | Strongly agree | Strongly agree | Strongly agree |
| Agree | Strongly agree | Agree | Agree |
| Agree | Strongly agree | Agree | Agree |
| Strongly agree | Strongly agree | Strongly agree | Strongly agree |
| Agree | Agree | Agree | Neutral |
| Strongly agree | Agree | Neutral | Neutral |
| Neutral | Strongly disagree | Agree | Disagree |
| Agree | Agree | Neutral | Neutral |
| Strongly agree | Strongly agree | Strongly agree | Strongly agree |
| Strongly disagree | Strongly disagree | Strongly disagree | Strongly disagree |
| Neutral | Neutral | Neutral | Neutral |
| Strongly agree | Strongly agree | Agree | Strongly agree |
| Neutral | Disagree | Neutral | Disagree |
| Agree | Agree | Neutral | Agree |
| Strongly disagree | Strongly disagree | Strongly disagree | Neutral |
| Strongly agree | Strongly agree | Strongly agree | Strongly agree |
| Strongly agree | Strongly agree | Strongly agree | Strongly agree |
| Strongly disagree | Strongly disagree | Neutral | Strongly disagree |
| Strongly disagree | Strongly disagree | Strongly disagree | Disagree |
| Strongly agree | Agree | Agree | Strongly agree |
| Agree | Strongly agree | Agree | Agree |
| Strongly agree | Strongly agree | Strongly agree | Strongly agree |
| Agree | Agree | Agree | Agree |
| Strongly agree | Strongly agree | Strongly agree | Strongly agree |
| Agree | Agree | Agree | Agree |
| Neutral | Disagree | Agree | Agree |
| Agree | Agree | Agree | Agree |
| Strongly agree | Strongly agree | Strongly agree | Strongly agree |
| Strongly agree | Strongly agree | Strongly agree | Strongly agree |
| Neutral | Agree | Strongly agree | Strongly agree |

| | | | |
|---|---|---|---|
| Strongly agree | Strongly agree | Agree | Agree |
| Strongly disagree | Strongly disagree | Strongly disagree | Strongly disagree |
| Strongly agree | Strongly agree | Agree | Strongly agree |
| Agree | Strongly agree | Neutral | Agree |
| Agree | Agree | Agree | Agree |
| Agree | Agree | Neutral | Neutral |
| Strongly agree | Strongly agree | Strongly agree | Strongly agree |
| Strongly agree | Agree | Agree | Agree |
| Strongly agree | Strongly agree | Strongly agree | Strongly agree |
| Strongly agree | Strongly agree | Strongly agree | Neutral |
| Strongly disagree | Strongly disagree | Strongly disagree | Strongly disagree |
| Agree | Agree | Agree | Agree |
| Strongly disagree | Strongly disagree | Strongly disagree | Strongly disagree |
| Agree | Agree | Agree | Agree |
| Strongly agree | Strongly agree | Strongly agree | Strongly agree |
| Strongly agree | Agree | Agree | Agree |
| Agree | Strongly agree | Agree | Agree |
| Strongly agree | Strongly agree | Strongly agree | Strongly agree |
| Agree | Agree | Neutral | Agree |
| Neutral | Agree | Neutral | Agree |
| Agree | Strongly agree | Strongly agree | Strongly agree |
| Agree | Agree | Agree | Agree |
| Strongly agree | Strongly agree | Strongly agree | Strongly agree |
| Strongly agree | Strongly agree | Agree | Strongly agree |
| Agree | Neutral | Neutral | Agree |
| Strongly agree | Strongly agree | Agree | Neutral |
| Agree | Agree | Neutral | Agree |
| Strongly agree | Strongly agree | Strongly agree | Strongly agree |
| Strongly agree | Strongly agree | Strongly agree | Strongly agree |
| Strongly agree | Strongly agree | Agree | Agree |
| Agree | Agree | Neutral | Agree |
| Strongly agree | Strongly agree | Strongly agree | Strongly agree |
| Agree | Agree | Agree | Agree |
| Strongly agree | Strongly agree | Disagree | Agree |
| Strongly agree | Strongly agree | Strongly agree | Strongly agree |
| Agree | Strongly agree | Strongly agree | Agree |
| Strongly agree | Strongly agree | Strongly agree | Agree |
| Agree | Strongly agree | Neutral | Agree |
| Strongly agree | Strongly agree | Strongly agree | Disagree |
| Neutral | Agree | Neutral | Neutral |
| Strongly agree | Disagree | Disagree | Disagree |
| Neutral | Agree | Agree | Neutral |
| Disagree | Strongly agree | Strongly agree | Agree |
| Agree | Agree | Agree | Strongly agree |
| Agree | Strongly agree | Agree | Strongly agree |
| Agree | Agree | Agree | Agree |
| Strongly agree | Neutral | Neutral | Neutral |
| Strongly agree | Strongly agree | Strongly agree | Strongly agree |
| Strongly agree | Agree | Agree | Strongly agree |
| Strongly agree | Strongly agree | Strongly agree | Strongly agree |
| Agree | Strongly agree | Agree | Strongly agree |
| Strongly agree | Strongly agree | Strongly agree | Neutral |
| Strongly agree | Strongly agree | Strongly agree | Strongly agree |
| Strongly agree | Strongly agree | Strongly agree | Strongly agree |
| Agree | Agree | Strongly agree | Agree |
| Strongly agree | Strongly agree | Strongly agree | Strongly agree |
| Strongly agree | Strongly agree | Neutral | Agree |
| Disagree | Disagree | Disagree | Strongly disagree |
| Neutral | Strongly agree | Agree | Neutral |

| | | | |
|---|---|---|---|
| Strongly agree | Strongly agree | Agree | Strongly agree |
| Strongly agree | Strongly agree | Strongly agree | Agree |
| Strongly agree | Strongly agree | Strongly agree | Strongly agree |
| Neutral | Agree | Agree | Disagree |
| Strongly agree | Strongly agree | Strongly agree | Disagree |
| Strongly agree | Agree | Agree | Agree |
| Agree | Agree | Agree | Agree |
| Neutral | Strongly agree | Strongly agree | Strongly agree |
| Strongly agree | Agree | Neutral | Neutral |
| Strongly agree | Agree | Strongly agree | Neutral |
| Agree | Strongly agree | Agree | Strongly agree |
| Strongly agree | Strongly agree | Agree | Neutral |
| Strongly agree | Strongly agree | Agree | Strongly agree |
| Strongly agree | Strongly agree | Strongly agree | Strongly agree |
| Agree | Agree | Agree | Agree |
| Strongly agree | Strongly agree | Strongly agree | Strongly agree |
| Agree | Agree | Disagree | Agree |
| Strongly agree | Strongly agree | Neutral | Neutral |
| Strongly agree | Agree | Strongly agree | Neutral |
| Strongly agree | Strongly agree | Strongly agree | Strongly agree |
| Strongly agree | Strongly agree | Strongly agree | Strongly agree |
| Agree | Agree | Agree | Strongly agree |
| Agree | Disagree | Agree | Agree |
| Strongly agree | Strongly agree | Agree | Strongly agree |
| Strongly agree | Strongly agree | Strongly agree | Strongly agree |
| Strongly agree | Strongly agree | Agree | Strongly agree |
| Strongly agree | Strongly agree | Neutral | Strongly agree |
| Strongly agree | Agree | Strongly agree | Strongly agree |
| Strongly agree | Strongly agree | Strongly agree | Strongly agree |
| Neutral | Strongly agree | Neutral | Agree |
| Agree | Agree | Agree | Agree |
| Strongly disagree | Agree | Neutral | Agree |
| Strongly agree | Strongly agree | Agree | Strongly agree |
| Agree | Neutral | Neutral | Neutral |
| Strongly agree | Strongly agree | Neutral | Agree |
| Agree | Agree | Neutral | Agree |
| Neutral | Neutral | Neutral | Neutral |
| Strongly agree | Agree | Neutral | Strongly agree |
| Agree | Agree | Agree | Agree |
| Agree | Agree | Agree | Neutral |
| Strongly agree | Strongly agree | Strongly agree | Agree |
| Neutral | Neutral | Neutral | Neutral |
| Agree | Agree | Agree | Strongly agree |
| Strongly agree | Strongly agree | Strongly agree | Strongly agree |
| Agree | Agree | Strongly agree | Agree |
| Strongly disagree | Neutral | Disagree | Disagree |
| Strongly disagree | Strongly disagree | Disagree | Disagree |
| Strongly agree | Agree | Agree | Agree |
| Strongly agree | Strongly agree | Strongly agree | Strongly agree |
| Neutral | Strongly agree | Disagree | Strongly agree |
| Neutral | Agree | Disagree | Agree |
| Agree | Strongly agree | Strongly agree | Agree |
| Agree | Strongly agree | Neutral | Neutral |
| Strongly agree | Strongly agree | Agree | Strongly agree |
| Strongly agree | Strongly agree | Strongly agree | Strongly agree |
| Strongly agree | Strongly agree | Strongly agree | Strongly agree |
| Agree | Disagree | Neutral | Neutral |
| Strongly agree | Strongly agree | Strongly agree | Strongly agree |
| Neutral | Strongly agree | Strongly agree | Neutral |

| | | | |
|---|---|---|---|
| Strongly agree | Strongly agree | Strongly agree | Strongly agree |
| Agree | Strongly agree | Agree | Neutral |
| Agree | Agree | Agree | Agree |
| Strongly agree | Neutral | Neutral | Agree |
| Agree | Agree | Neutral | Agree |
| Agree | Strongly agree | Agree | Strongly agree |
| Agree | Disagree | Agree | Agree |
| Strongly agree | Strongly agree | Strongly agree | Strongly agree |
| Agree | Agree | Agree | Agree |
| Agree | Disagree | Neutral | Disagree |
| Agree | Agree | Strongly agree | Strongly agree |
| Agree | Strongly agree | Agree | Strongly agree |
| Agree | Agree | Agree | Strongly agree |
| Strongly agree | Agree | Agree | Strongly agree |
| Strongly agree | Agree | Strongly agree | Agree |
| Neutral | Neutral | Neutral | Neutral |
| Strongly agree | Strongly agree | Agree | Strongly agree |
| Strongly agree | Strongly agree | Strongly agree | Strongly agree |
| Disagree | Disagree | Strongly agree | Strongly disagree |
| Strongly agree | Strongly agree | Strongly agree | Strongly agree |
| Agree | Agree | Agree | Agree |
| Strongly agree | Strongly agree | Strongly agree | Strongly agree |
| Strongly agree | Strongly agree | Strongly agree | Strongly agree |

| V6- Shared learning wil | V7- For small group lea | V8-Team-working skills | V9- Shared learning wil |
|---|---|---|---|
| Agree | Strongly agree | Agree | Strongly agree |
| Strongly agree | Strongly agree | Strongly agree | Strongly agree |
| Neutral | Neutral | Neutral | Neutral |
| Strongly disagree | Strongly disagree | Disagree | Strongly disagree |
| Agree | Agree | Agree | Neutral |
| Strongly agree | Strongly agree | Strongly agree | Strongly agree |
| Agree | Strongly agree | Strongly agree | Agree |
| Neutral | Neutral | Neutral | Neutral |
| Strongly disagree | Strongly disagree | Strongly disagree | Agree |
| Neutral | Strongly agree | Agree | Agree |
| Strongly agree | Disagree | Strongly disagree | Strongly disagree |
| Agree | Strongly agree | Strongly agree | Neutral |
| Strongly agree | Strongly agree | Strongly agree | Strongly agree |
| Strongly disagree | Strongly disagree | Disagree | Neutral |
| Agree | Agree | Agree | Agree |
| Agree | Strongly agree | Strongly agree | Strongly agree |
| Neutral | Strongly agree | Strongly agree | Neutral |
| Strongly agree | Strongly agree | Strongly agree | Strongly agree |
| Agree | Agree | Agree | Disagree |
| Neutral | Strongly agree | Strongly agree | Agree |
| Strongly disagree | Strongly disagree | Strongly disagree | Strongly disagree |
| Agree | Agree | Agree | Agree |
| Neutral | Neutral | Strongly agree | Neutral |
| Strongly agree | Strongly agree | Strongly agree | Strongly agree |
| Strongly agree | Strongly agree | Agree | Strongly agree |
| Agree | Strongly agree | Neutral | Strongly agree |
| Neutral | Neutral | Strongly agree | Agree |
| Agree | Agree | Agree | Neutral |
| Strongly disagree | Strongly disagree | Strongly disagree | Strongly disagree |
| Disagree | Strongly agree | Strongly disagree | Disagree |
| Agree | Strongly agree | Agree | Strongly agree |
| Strongly agree | Strongly agree | Strongly agree | Agree |
| Strongly agree | Strongly agree | Strongly agree | Strongly agree |
| Agree | Strongly agree | Strongly agree | Agree |
| Neutral | Strongly agree | Agree | Strongly agree |
| Strongly agree | Strongly agree | Strongly agree | Strongly agree |
| Strongly agree | Agree | Neutral | Neutral |
| Strongly agree | Strongly agree | Strongly agree | Strongly agree |
| Strongly agree | Strongly agree | Strongly agree | Strongly agree |
| Agree | Strongly agree | Strongly agree | Agree |
| Agree | Agree | Agree | Agree |
| Neutral | Strongly agree | Agree | Strongly agree |
| Agree | Agree | Strongly agree | Agree |
| Strongly agree | Strongly agree | Strongly agree | Strongly agree |
| Strongly agree | Agree | Neutral | Agree |
| Neutral | Neutral | Neutral | Neutral |
| Strongly agree | Agree | Strongly agree | Agree |
| Agree | Strongly agree | Neutral | Agree |
| Strongly agree | Strongly agree | Agree | Strongly agree |
| Agree | Agree | Disagree | Agree |
| Strongly agree | Strongly agree | Strongly agree | Neutral |
| Neutral | Strongly agree | Strongly agree | Neutral |

| | | | |
|---|---|---|---|
| Strongly agree | Strongly agree | Strongly agree | Strongly agree |
| Strongly agree | Agree | Agree | Strongly agree |
| Agree | Agree | Agree | Agree |
| Strongly disagree | Disagree | Agree | Strongly agree |
| Agree | Strongly agree | Agree | Neutral |
| Neutral | Strongly agree | Neutral | Strongly agree |
| Neutral | Neutral | Neutral | Neutral |
| Neutral | Neutral | Neutral | Neutral |
| Strongly agree | Strongly agree | Strongly agree | Strongly agree |
| Strongly agree | Strongly agree | Strongly agree | Strongly agree |
| Disagree | Disagree | Disagree | Disagree |
| Agree | Strongly agree | Strongly agree | Agree |
| Agree | Strongly agree | Agree | Strongly agree |
| Disagree | Agree | Neutral | Neutral |
| Neutral | Strongly agree | Agree | Agree |
| Strongly agree | Strongly agree | Strongly agree | Strongly agree |
| Agree | Strongly agree | Strongly agree | Strongly agree |
| Strongly agree | Strongly agree | Neutral | Agree |
| Agree | Strongly agree | Strongly agree | Agree |
| Strongly agree | Strongly agree | Strongly agree | Strongly agree |
| Strongly agree | Strongly agree | Neutral | Strongly disagree |
| Strongly agree | Strongly agree | Strongly agree | Strongly agree |
| Agree | Agree | Agree | Agree |
| Agree | Agree | Neutral | Agree |
| Neutral | Agree | Neutral | Neutral |
| Neutral | Strongly agree | Strongly agree | Neutral |
| Agree | Strongly agree | Strongly agree | Strongly agree |
| Strongly agree | Strongly agree | Strongly agree | Strongly agree |
| Agree | Strongly agree | Strongly agree | Agree |
| Disagree | Strongly agree | Disagree | Neutral |
| Neutral | Strongly agree | Neutral | Neutral |
| Strongly agree | Strongly agree | Strongly agree | Strongly agree |
| Neutral | Strongly agree | Strongly agree | Agree |
| Agree | Agree | Agree | Agree |
| Agree | Agree | Agree | Agree |
| Neutral | Disagree | Strongly disagree | Disagree |
| Agree | Agree | Agree | Neutral |
| Agree | Strongly agree | Agree | Neutral |
| Neutral | Strongly agree | Neutral | Disagree |
| Agree | Agree | Agree | Agree |
| Neutral | Agree | Strongly agree | Agree |
| Neutral | Strongly agree | Agree | Neutral |
| Agree | Agree | Agree | Agree |
| Strongly agree | Strongly agree | Strongly agree | Strongly agree |
| Agree | Strongly agree | Agree | Agree |
| Agree | Strongly agree | Strongly agree | Strongly agree |
| Strongly agree | Agree | Agree | Agree |
| Neutral | Strongly agree | Agree | Neutral |
| Agree | Agree | Strongly agree | Neutral |
| Agree | Strongly agree | Strongly agree | Agree |
| Strongly agree | Strongly agree | Agree | Agree |
| Agree | Strongly agree | Agree | Agree |
| Agree | Agree | Agree | Agree |
| Strongly agree | Strongly agree | Strongly agree | Strongly agree |
| Agree | Strongly agree | Neutral | Neutral |
| Agree | Strongly agree | Strongly agree | Neutral |
| Agree | Agree | Agree | Agree |
| Neutral | Neutral | Neutral | Neutral |
| Strongly agree | Strongly agree | Strongly agree | Agree |

| | | | |
|---|---|---|---|
| Agree | Agree | Agree | Agree |
| Strongly agree | Strongly agree | Strongly agree | Strongly agree |
| Neutral | Strongly agree | Strongly agree | Agree |
| Agree | Agree | Agree | Agree |
| Strongly agree | Strongly agree | Strongly agree | Strongly agree |
| Agree | Agree | Disagree | Neutral |
| Strongly agree | Strongly agree | Strongly agree | Strongly agree |
| Neutral | Neutral | Neutral | Agree |
| Strongly disagree | Strongly disagree | Strongly disagree | Strongly disagree |
| Strongly agree | Agree | Neutral | Strongly agree |
| Agree | Agree | Agree | Agree |
| Agree | Agree | Agree | Agree |
| Neutral | Neutral | Neutral | Neutral |
| Agree | Strongly agree | Agree | Strongly agree |
| Strongly agree | Strongly agree | Neutral | Disagree |
| Agree | Agree | Neutral | Agree |
| Agree | Agree | Agree | Agree |
| Strongly agree | Strongly agree | Strongly agree | Strongly agree |
| Neutral | Agree | Strongly agree | Neutral |
| Strongly agree | Strongly agree | Strongly agree | Strongly agree |
| Strongly agree | Strongly agree | Strongly agree | Strongly agree |
| Agree | Strongly agree | Agree | Strongly agree |
| Agree | Strongly agree | Strongly agree | Agree |
| Agree | Neutral | Strongly agree | Neutral |
| Strongly agree | Strongly agree | Strongly agree | Strongly agree |
| Strongly agree | Agree | Agree | Agree |
| Strongly agree | Strongly agree | Strongly agree | Strongly agree |
| Strongly agree | Strongly agree | Strongly agree | Strongly agree |
| Strongly agree | Strongly agree | Strongly agree | Strongly agree |
| Strongly agree | Strongly agree | Strongly agree | Agree |
| Agree | Agree | Agree | Agree |
| Agree | Agree | Agree | Agree |
| Strongly agree | Strongly agree | Strongly agree | Strongly agree |
| Agree | Strongly agree | Agree | Agree |
| Neutral | Neutral | Neutral | Neutral |
| Neutral | Strongly agree | Agree | Agree |
| Neutral | Neutral | Agree | Strongly agree |
| Strongly agree | Strongly agree | Strongly agree | Strongly agree |
| Disagree | Strongly disagree | Disagree | Strongly disagree |
| Neutral | Neutral | Strongly agree | Neutral |
| Strongly agree | Strongly agree | Strongly agree | Strongly agree |
| Disagree | Agree | Agree | Neutral |
| Disagree | Agree | Agree | Disagree |
| Neutral | Neutral | Neutral | Neutral |
| Strongly agree | Strongly agree | Strongly agree | Strongly agree |
| Strongly agree | Strongly agree | Strongly agree | Strongly agree |
| Strongly disagree | Neutral | Disagree | Agree |
| Strongly disagree | Disagree | Strongly disagree | Strongly disagree |
| Agree | Strongly agree | Strongly agree | Agree |
| Strongly agree | Strongly agree | Strongly agree | Strongly agree |
| Strongly agree | Strongly agree | Strongly agree | Strongly agree |
| Agree | Agree | Agree | Neutral |
| Strongly agree | Strongly agree | Strongly agree | Strongly agree |
| Agree | Agree | Agree | Agree |
| Disagree | Neutral | Disagree | Neutral |
| Agree | Agree | Agree | Agree |
| Strongly agree | Strongly agree | Strongly agree | Strongly agree |
| Strongly agree | Strongly agree | Strongly agree | Strongly agree |
| Agree | Strongly agree | Strongly agree | Strongly agree |

| | | | |
|---|---|---|---|
| Strongly agree | Strongly agree | Agree | Strongly agree |
| Strongly disagree | Strongly disagree | Strongly disagree | Strongly disagree |
| Strongly disagree | Strongly agree | Strongly disagree | Strongly agree |
| Agree | Agree | Strongly agree | Agree |
| Agree | Agree | Agree | Agree |
| Neutral | Agree | Agree | Neutral |
| Strongly agree | Strongly agree | Strongly agree | Strongly agree |
| Agree | Agree | Strongly agree | Agree |
| Strongly agree | Strongly agree | Strongly agree | Strongly agree |
| Strongly agree | Strongly agree | Strongly agree | Strongly agree |
| Strongly disagree | Strongly disagree | Strongly disagree | Strongly disagree |
| Agree | Agree | Agree | Agree |
| Strongly disagree | Strongly disagree | Strongly disagree | Strongly disagree |
| Agree | Agree | Agree | Agree |
| Strongly agree | Strongly agree | Strongly agree | Strongly agree |
| Agree | Agree | Agree | Agree |
| Agree | Strongly agree | Strongly agree | Strongly agree |
| Strongly agree | Strongly agree | Strongly agree | Strongly agree |
| Agree | Strongly agree | Agree | Agree |
| Agree | Agree | Agree | Agree |
| Strongly agree | Strongly agree | Strongly agree | Strongly agree |
| Agree | Strongly agree | Strongly agree | Agree |
| Strongly agree | Strongly agree | Strongly agree | Strongly agree |
| Strongly agree | Strongly agree | Strongly agree | Strongly agree |
| Agree | Agree | Agree | Neutral |
| Strongly agree | Strongly agree | Agree | Strongly agree |
| Strongly agree | Agree | Strongly agree | Agree |
| Strongly agree | Strongly agree | Strongly agree | Strongly agree |
| Strongly agree | Strongly agree | Strongly agree | Strongly agree |
| Strongly agree | Strongly agree | Strongly agree | Agree |
| Agree | Agree | Agree | Agree |
| Strongly agree | Strongly agree | Strongly agree | Agree |
| Neutral | Strongly agree | Agree | Neutral |
| Disagree | Agree | Agree | Strongly agree |
| Strongly agree | Strongly agree | Strongly agree | Strongly agree |
| Agree | Strongly agree | Strongly agree | Disagree |
| Agree | Strongly agree | Agree | Agree |
| Strongly agree | Strongly agree | Strongly agree | Neutral |
| Strongly agree | Strongly agree | Strongly agree | Strongly agree |
| Agree | Strongly disagree | Strongly agree | Disagree |
| Strongly disagree | Strongly disagree | Disagree | Strongly disagree |
| Agree | Strongly agree | Agree | Agree |
| Neutral | Strongly agree | Strongly agree | Agree |
| Strongly agree | Strongly agree | Strongly agree | Strongly agree |
| Strongly agree | Strongly agree | Agree | Agree |
| Agree | Agree | Strongly agree | Strongly agree |
| Agree | Agree | Agree | Neutral |
| Agree | Strongly agree | Agree | Neutral |
| Strongly agree | Strongly agree | Strongly agree | Strongly agree |
| Agree | Strongly agree | Strongly agree | Strongly agree |
| Strongly agree | Strongly agree | Strongly agree | Agree |
| Strongly agree | Strongly agree | Strongly agree | Strongly agree |
| Strongly agree | Strongly agree | Strongly agree | Strongly agree |
| Agree | Strongly agree | Strongly agree | Strongly agree |
| Strongly agree | Agree | Strongly agree | Strongly agree |
| Strongly agree | Strongly agree | Strongly agree | Agree |
| Agree | Strongly agree | Strongly agree | Strongly agree |
| Disagree | Disagree | Neutral | Neutral |
| Strongly agree | Strongly agree | Disagree | Agree |

| | | | |
|---|---|---|---|
| Agree | Strongly agree | Agree | Agree |
| Agree | Strongly agree | Agree | Strongly agree |
| Agree | Strongly agree | Strongly agree | Disagree |
| Agree | Strongly disagree | Agree | Strongly agree |
| Strongly agree | Strongly agree | Strongly agree | Strongly agree |
| Agree | Strongly agree | Strongly agree | Agree |
| Agree | Agree | Agree | Neutral |
| Neutral | Strongly agree | Strongly agree | Agree |
| Neutral | Agree | Strongly agree | Neutral |
| Strongly agree | Strongly agree | Agree | Strongly agree |
| Agree | Agree | Agree | Agree |
| Agree | Strongly agree | Agree | Agree |
| Strongly agree | Strongly agree | Strongly agree | Strongly agree |
| Strongly agree | Strongly agree | Strongly agree | Strongly agree |
| Agree | Agree | Agree | Agree |
| Strongly agree | Strongly agree | Strongly agree | Strongly agree |
| Agree | Agree | Agree | Agree |
| Strongly agree | Agree | Strongly agree | Neutral |
| Agree | Strongly agree | Strongly agree | Agree |
| Strongly agree | Strongly agree | Strongly agree | Strongly agree |
| Strongly agree | Strongly agree | Strongly agree | Strongly agree |
| Agree | Strongly agree | Strongly agree | Strongly agree |
| Agree | Neutral | Disagree | Neutral |
| Strongly agree | Strongly agree | Strongly agree | Agree |
| Strongly agree | Strongly agree | Strongly agree | Strongly agree |
| Strongly agree | Strongly agree | Strongly agree | Strongly agree |
| Strongly agree | Strongly agree | Strongly agree | Strongly agree |
| Strongly agree | Strongly agree | Strongly agree | Agree |
| Strongly agree | Strongly agree | Strongly agree | Strongly agree |
| Strongly agree | Strongly agree | Strongly agree | Strongly disagree |
| Agree | Strongly agree | Strongly agree | Neutral |
| Agree | Strongly agree | Agree | Agree |
| Agree | Strongly agree | Strongly agree | Agree |
| Agree | Agree | Agree | Agree |
| Agree | Agree | Agree | Agree |
| Agree | Agree | Neutral | Agree |
| Neutral | Neutral | Neutral | Neutral |
| Agree | Neutral | Strongly agree | Agree |
| Agree | Agree | Agree | Neutral |
| Agree | Strongly agree | Strongly agree | Agree |
| Strongly agree | Strongly agree | Strongly agree | Agree |
| Neutral | Neutral | Neutral | Neutral |
| Agree | Agree | Agree | Agree |
| Strongly agree | Strongly agree | Strongly agree | Strongly agree |
| Neutral | Strongly agree | Strongly agree | Agree |
| Disagree | Disagree | Disagree | Disagree |
| Disagree | Strongly disagree | Strongly disagree | Strongly disagree |
| Neutral | Disagree | Strongly disagree | Neutral |
| Strongly agree | Strongly agree | Strongly agree | Strongly agree |
| Strongly agree | Strongly agree | Agree | Strongly agree |
| Agree | Agree | Agree | Agree |
| Agree | Strongly agree | Strongly agree | Agree |
| Agree | Agree | Agree | Agree |
| Neutral | Strongly agree | Strongly agree | Strongly agree |
| Strongly agree | Strongly agree | Strongly agree | Strongly agree |
| Strongly agree | Strongly agree | Strongly agree | Strongly agree |
| Neutral | Agree | Agree | Agree |
| Strongly agree | Strongly agree | Strongly agree | Strongly agree |
| Strongly disagree | Strongly agree | Neutral | Strongly agree |

| | | | |
|---|---|---|---|
| Strongly agree | Strongly agree | Strongly agree | Neutral |
| Strongly agree | Strongly agree | Strongly agree | Strongly agree |
| Agree | Strongly agree | Agree | Agree |
| Disagree | Agree | Disagree | Disagree |
| Agree | Agree | Neutral | Neutral |
| Agree | Strongly agree | Agree | Agree |
| Neutral | Strongly agree | Strongly agree | Agree |
| Strongly agree | Strongly agree | Strongly agree | Strongly agree |
| Agree | Agree | Strongly agree | Agree |
| Neutral | Strongly agree | Strongly disagree | Disagree |
| Agree | Strongly agree | Agree | Agree |
| Strongly agree | Strongly agree | Agree | Strongly agree |
| Strongly agree | Strongly agree | Strongly agree | Strongly agree |
| Strongly agree | Strongly agree | Strongly agree | Strongly agree |
| Agree | Strongly agree | Strongly agree | Strongly agree |
| Neutral | Neutral | Neutral | Neutral |
| Strongly agree | Strongly agree | Strongly agree | Strongly agree |
| Strongly agree | Strongly agree | Strongly agree | Strongly agree |
| Disagree | Agree | Strongly disagree | Strongly agree |
| Strongly agree | Strongly agree | Strongly agree | Strongly agree |
| Agree | Agree | Agree | Agree |
| Strongly agree | Strongly agree | Strongly agree | Strongly agree |
| Strongly agree | Strongly agree | Strongly agree | Strongly agree |

| V10- I don't want to wa: | V11- It is not necessary | V12- Clinical problem-s | V13- Shared learning w |
|---|---|---|---|
| Disagree | Disagree | Neutral | Neutral |
| Neutral | Disagree | Disagree | Strongly agree |
| Neutral | Neutral | Neutral | Neutral |
| Disagree | Strongly agree | Strongly disagree | Strongly disagree |
| Agree | Strongly agree | Neutral | Disagree |
| Strongly disagree | Strongly disagree | Disagree | Strongly agree |
| Disagree | Disagree | Agree | Neutral |
| Neutral | Neutral | Neutral | Neutral |
| Disagree | Disagree | Disagree | Strongly disagree |
| Disagree | Strongly disagree | Strongly disagree | Strongly agree |
| Agree | Strongly agree | Strongly disagree | Neutral |
| Disagree | Disagree | Agree | Agree |
| Neutral | Strongly agree | Strongly agree | Strongly agree |
| Strongly agree | Agree | Neutral | Disagree |
| Disagree | Disagree | Neutral | Neutral |
| Strongly disagree | Strongly disagree | Strongly disagree | Strongly agree |
| Strongly agree | Strongly agree | Strongly disagree | Disagree |
| Strongly disagree | Strongly disagree | Disagree | Strongly agree |
| Disagree | Disagree | Disagree | Agree |
| Disagree | Disagree | Neutral | Agree |
| Strongly disagree | Strongly disagree | Strongly disagree | Strongly disagree |
| Disagree | Disagree | Disagree | Agree |
| Strongly disagree | Strongly disagree | Agree | Agree |
| Disagree | Strongly disagree | Disagree | Strongly agree |
| Disagree | Disagree | Disagree | Disagree |
| Strongly disagree | Neutral | Disagree | Strongly agree |
| Agree | Strongly agree | Strongly agree | Neutral |
| Neutral | Disagree | Disagree | Agree |
| Strongly disagree | Strongly disagree | Strongly disagree | Strongly disagree |
| Strongly agree | Disagree | Disagree | Agree |
| Strongly disagree | Strongly disagree | Neutral | Strongly agree |
| Disagree | Disagree | Neutral | Strongly agree |
| Strongly agree | Strongly disagree | Neutral | Neutral |
| Strongly disagree | Disagree | Neutral | Strongly agree |
| Strongly agree | Strongly agree | Strongly agree | Agree |
| Strongly disagree | Strongly disagree | Strongly disagree | Strongly agree |
| Agree | Strongly disagree | Agree | Neutral |
| Strongly agree | Strongly agree | Strongly agree | Strongly agree |
| Strongly disagree | Strongly disagree | Disagree | Strongly agree |
| Neutral | Neutral | Disagree | Neutral |
| Disagree | Neutral | Neutral | Agree |
| Disagree | Strongly disagree | Neutral | Neutral |
| Agree | Agree | Agree | Agree |
| Strongly disagree | Strongly agree | Strongly disagree | Strongly agree |
| Agree | Neutral | Strongly agree | Agree |
| Neutral | Neutral | Neutral | Neutral |
| Strongly disagree | Disagree | Disagree | Strongly agree |
| Agree | Strongly agree | Strongly agree | Disagree |
| Disagree | Neutral | Neutral | Agree |
| Neutral | Agree | Agree | Neutral |
| Strongly disagree | Strongly disagree | Neutral | Strongly agree |
| Disagree | Disagree | Disagree | Agree |

| | | | |
|---|---|---|---|
| Disagree | Neutral | Disagree | Strongly agree |
| Neutral | Disagree | Disagree | Agree |
| Disagree | Disagree | Neutral | Agree |
| Agree | Neutral | Strongly disagree | Strongly agree |
| Disagree | Disagree | Agree | Strongly agree |
| Neutral | Disagree | Neutral | Agree |
| Neutral | Neutral | Neutral | Neutral |
| Neutral | Neutral | Neutral | Neutral |
| Neutral | Disagree | Agree | Agree |
| Disagree | Strongly disagree | Strongly disagree | Strongly agree |
| Disagree | Disagree | Disagree | Disagree |
| Disagree | Strongly disagree | Neutral | Strongly agree |
| Agree | Disagree | Strongly disagree | Strongly agree |
| Neutral | Disagree | Neutral | Neutral |
| Neutral | Disagree | Disagree | Agree |
| Strongly disagree | Strongly disagree | Strongly disagree | Strongly agree |
| Neutral | Strongly disagree | Neutral | Strongly agree |
| Disagree | Strongly disagree | Disagree | Agree |
| Neutral | Disagree | Neutral | Agree |
| Strongly disagree | Neutral | Disagree | Neutral |
| Strongly disagree | Agree | Strongly agree | Disagree |
| Disagree | Disagree | Strongly disagree | Agree |
| Agree | Agree | Neutral | Agree |
| Neutral | Neutral | Agree | Agree |
| Agree | Neutral | Agree | Neutral |
| Strongly disagree | Strongly disagree | Strongly disagree | Strongly agree |
| Disagree | Disagree | Disagree | Strongly agree |
| Strongly disagree | Disagree | Disagree | Strongly agree |
| Strongly disagree | Strongly disagree | Disagree | Agree |
| Strongly agree | Strongly disagree | Strongly disagree | Strongly disagree |
| Neutral | Neutral | Strongly agree | Agree |
| Disagree | Strongly disagree | Strongly disagree | Strongly agree |
| Disagree | Disagree | Agree | Agree |
| Agree | Agree | Agree | Agree |
| Strongly agree | Agree | Agree | Agree |
| Strongly disagree | Disagree | Strongly disagree | Neutral |
| Disagree | Disagree | Agree | Neutral |
| Agree | Disagree | Neutral | Neutral |
| Neutral | Agree | Disagree | Strongly agree |
| Agree | Agree | Agree | Agree |
| Agree | Agree | Strongly agree | Strongly agree |
| Strongly disagree | Strongly disagree | Strongly disagree | Strongly agree |
| Strongly agree | Strongly agree | Neutral | Agree |
| Strongly disagree | Disagree | Neutral | Strongly agree |
| Disagree | Strongly disagree | Strongly disagree | Agree |
| Strongly disagree | Strongly disagree | Strongly agree | Strongly agree |
| Agree | Agree | Strongly agree | Agree |
| Disagree | Disagree | Neutral | Agree |
| Neutral | Agree | Strongly agree | Strongly agree |
| Disagree | Disagree | Agree | Neutral |
| Disagree | Disagree | Disagree | Agree |
| Disagree | Strongly disagree | Disagree | Agree |
| Agree | Agree | Agree | Agree |
| Strongly agree | Strongly agree | Strongly agree | Strongly agree |
| Strongly disagree | Strongly disagree | Strongly disagree | Agree |
| Agree | Disagree | Neutral | Agree |
| Agree | Disagree | Agree | Agree |
| Neutral | Neutral | Neutral | Neutral |
| Strongly disagree | Strongly disagree | Disagree | Agree |

| | | | |
|---|---|---|---|
| Disagree | Disagree | Disagree | Agree |
| Strongly disagree | Strongly disagree | Strongly disagree | Strongly agree |
| Disagree | Disagree | Disagree | Agree |
| Disagree | Agree | Disagree | Agree |
| Strongly disagree | Strongly disagree | Strongly agree | Strongly agree |
| Disagree | Neutral | Disagree | Neutral |
| Strongly agree | Strongly agree | Strongly agree | Strongly agree |
| Neutral | Neutral | Neutral | Neutral |
| Strongly disagree | Strongly disagree | Strongly disagree | Strongly disagree |
| Agree | Agree | Agree | Agree |
| Agree | Agree | Agree | Agree |
| Agree | Agree | Agree | Strongly agree |
| Agree | Agree | Disagree | Neutral |
| Disagree | Disagree | Strongly agree | Strongly agree |
| Disagree | Disagree | Disagree | Strongly agree |
| Agree | Agree | Agree | Agree |
| Neutral | Neutral | Neutral | Neutral |
| Strongly agree | Strongly agree | Strongly agree | Strongly agree |
| Disagree | Disagree | Disagree | Neutral |
| Strongly disagree | Strongly disagree | Strongly disagree | Strongly agree |
| Strongly disagree | Strongly disagree | Disagree | Strongly agree |
| Disagree | Disagree | Neutral | Agree |
| Neutral | Strongly agree | Disagree | Strongly agree |
| Strongly disagree | Strongly disagree | Disagree | Agree |
| Neutral | Neutral | Neutral | Neutral |
| Agree | Agree | Agree | Agree |
| Neutral | Agree | Strongly agree | Strongly agree |
| Strongly disagree | Strongly disagree | Neutral | Neutral |
| Strongly agree | Strongly disagree | Strongly disagree | Strongly agree |
| Disagree | Neutral | Neutral | Agree |
| Strongly disagree | Strongly disagree | Disagree | Agree |
| Neutral | Neutral | Disagree | Agree |
| Disagree | Disagree | Strongly agree | Strongly agree |
| Neutral | Neutral | Agree | Agree |
| Neutral | Neutral | Neutral | Agree |
| Agree | Neutral | Disagree | Agree |
| Disagree | Neutral | Disagree | Neutral |
| Strongly agree | Strongly agree | Strongly agree | Strongly agree |
| Agree | Agree | Neutral | Strongly disagree |
| Neutral | Neutral | Strongly disagree | Strongly disagree |
| Strongly disagree | Disagree | Disagree | Strongly agree |
| Neutral | Neutral | Neutral | Neutral |
| Disagree | Neutral | Disagree | Neutral |
| Neutral | Neutral | Neutral | Neutral |
| Strongly agree | Strongly agree | Strongly agree | Strongly agree |
| Strongly agree | Strongly disagree | Strongly disagree | Strongly agree |
| Strongly disagree | Neutral | Strongly disagree | Disagree |
| Strongly disagree | Strongly disagree | Strongly disagree | Strongly disagree |
| Strongly disagree | Strongly disagree | Strongly disagree | Strongly agree |
| Disagree | Neutral | Agree | Strongly agree |
| Strongly agree | Strongly agree | Strongly agree | Strongly agree |
| Disagree | Disagree | Disagree | Agree |
| Strongly agree | Strongly agree | Strongly agree | Strongly agree |
| Neutral | Neutral | Agree | Disagree |
| Neutral | Neutral | Agree | Agree |
| Agree | Agree | Agree | Agree |
| Strongly disagree | Strongly disagree | Disagree | Strongly agree |
| Strongly disagree | Strongly disagree | Strongly disagree | Strongly agree |
| Disagree | Strongly disagree | Strongly disagree | Strongly agree |

| | | | |
|---|---|---|---|
| Disagree | Disagree | Strongly disagree | Strongly agree |
| Strongly disagree | Strongly disagree | Strongly disagree | Disagree |
| Disagree | Strongly agree | Neutral | Strongly agree |
| Disagree | Disagree | Neutral | Strongly agree |
| Neutral | Disagree | Disagree | Agree |
| Disagree | Neutral | Disagree | Neutral |
| Strongly disagree | Strongly disagree | Neutral | Strongly agree |
| Disagree | Strongly disagree | Disagree | Agree |
| Disagree | Strongly disagree | Agree | Strongly agree |
| Neutral | Disagree | Neutral | Agree |
| Strongly disagree | Strongly disagree | Strongly disagree | Strongly disagree |
| Agree | Agree | Agree | Agree |
| Strongly disagree | Strongly disagree | Strongly disagree | Strongly disagree |
| Agree | Agree | Agree | Agree |
| Strongly agree | Strongly agree | Strongly agree | Strongly agree |
| Disagree | Disagree | Neutral | Agree |
| Strongly agree | Strongly agree | Agree | Agree |
| Strongly agree | Strongly agree | Strongly agree | Strongly agree |
| Neutral | Neutral | Disagree | Agree |
| Neutral | Disagree | Disagree | Agree |
| Disagree | Disagree | Neutral | Strongly agree |
| Disagree | Disagree | Disagree | Agree |
| Strongly agree | Strongly agree | Strongly agree | Strongly agree |
| Strongly disagree | Strongly disagree | Strongly disagree | Strongly agree |
| Disagree | Disagree | Neutral | Agree |
| Strongly agree | Disagree | Disagree | Strongly agree |
| Disagree | Strongly disagree | Strongly disagree | Agree |
| Strongly disagree | Strongly disagree | Strongly disagree | Strongly agree |
| Strongly agree | Strongly agree | Strongly agree | Strongly agree |
| Strongly disagree | Disagree | Disagree | Strongly agree |
| Disagree | Agree | Neutral | Agree |
| Neutral | Neutral | Disagree | Strongly agree |
| Disagree | Neutral | Disagree | Strongly agree |
| Strongly disagree | Strongly disagree | Disagree | Agree |
| Disagree | Strongly disagree | Strongly disagree | Strongly agree |
| Strongly disagree | Disagree | Agree | Agree |
| Strongly agree | Agree | Agree | Strongly agree |
| Agree | Neutral | Neutral | Agree |
| Strongly disagree | Strongly disagree | Strongly disagree | Strongly agree |
| Neutral | Strongly agree | Strongly disagree | Agree |
| Strongly disagree | Strongly disagree | Disagree | Strongly disagree |
| Disagree | Neutral | Disagree | Neutral |
| Strongly disagree | Disagree | Strongly disagree | Strongly agree |
| Strongly disagree | Strongly disagree | Disagree | Strongly agree |
| Neutral | Neutral | Disagree | Agree |
| Strongly disagree | Strongly disagree | Strongly disagree | Strongly disagree |
| Neutral | Agree | Neutral | Neutral |
| Disagree | Strongly agree | Strongly agree | Strongly agree |
| Strongly disagree | Strongly disagree | Strongly disagree | Strongly agree |
| Strongly agree | Strongly agree | Strongly agree | Strongly agree |
| Disagree | Disagree | Neutral | Strongly agree |
| Disagree | Neutral | Disagree | Agree |
| Strongly disagree | Strongly disagree | Strongly agree | Strongly agree |
| Disagree | Strongly disagree | Disagree | Strongly agree |
| Strongly disagree | Strongly disagree | Neutral | Strongly agree |
| Neutral | Disagree | Disagree | Strongly agree |
| Strongly disagree | Strongly disagree | Agree | Agree |
| Neutral | Neutral | Neutral | Neutral |
| Strongly agree | Agree | Agree | Agree |

| | | | |
|---|---|---|---|
| Strongly disagree | Strongly disagree | Strongly disagree | Strongly agree |
| Strongly agree | Disagree | Disagree | Agree |
| Strongly disagree | Strongly disagree | Strongly disagree | Strongly agree |
| Agree | Strongly agree | Agree | Agree |
| Strongly disagree | Strongly disagree | Strongly agree | Strongly agree |
| Strongly disagree | Strongly disagree | Disagree | Strongly agree |
| Disagree | Neutral | Disagree | Agree |
| Disagree | Neutral | Agree | Strongly agree |
| Disagree | Strongly disagree | Strongly disagree | Agree |
| Disagree | Strongly disagree | Neutral | Strongly agree |
| Disagree | Disagree | Neutral | Agree |
| Disagree | Disagree | Disagree | Strongly agree |
| Strongly disagree | Strongly disagree | Strongly disagree | Strongly agree |
| Disagree | Disagree | Strongly disagree | Strongly agree |
| Agree | Agree | Agree | Agree |
| Strongly disagree | Strongly disagree | Disagree | Strongly agree |
| Neutral | Disagree | Disagree | Agree |
| Disagree | Disagree | Disagree | Agree |
| Strongly disagree | Strongly disagree | Strongly disagree | Strongly agree |
| Disagree | Strongly disagree | Strongly disagree | Agree |
| Disagree | Neutral | Strongly disagree | Strongly agree |
| Strongly disagree | Strongly disagree | Strongly disagree | Strongly agree |
| Agree | Neutral | Disagree | Strongly agree |
| Strongly disagree | Disagree | Disagree | Strongly agree |
| Neutral | Disagree | Neutral | Agree |
| Disagree | Strongly disagree | Neutral | Strongly agree |
| Strongly agree | Strongly disagree | Neutral | Strongly agree |
| Strongly disagree | Disagree | Strongly disagree | Strongly agree |
| Disagree | Disagree | Disagree | Agree |
| Disagree | Strongly disagree | Neutral | Strongly agree |
| Strongly disagree | Strongly disagree | Disagree | Strongly agree |
| Strongly agree | Neutral | Strongly agree | Neutral |
| Strongly disagree | Strongly disagree | Disagree | Strongly agree |
| Agree | Neutral | Neutral | Agree |
| Disagree | Strongly disagree | Strongly disagree | Strongly agree |
| Neutral | Agree | Neutral | Strongly agree |
| Neutral | Neutral | Neutral | Neutral |
| Neutral | Agree | Strongly agree | Agree |
| Disagree | Disagree | Neutral | Strongly agree |
| Disagree | Disagree | Strongly disagree | Strongly agree |
| Disagree | Strongly disagree | Disagree | Strongly agree |
| Neutral | Neutral | Neutral | Neutral |
| Neutral | Neutral | Agree | Agree |
| Strongly disagree | Neutral | Neutral | Strongly agree |
| Neutral | Neutral | Neutral | Agree |
| Strongly disagree | Strongly disagree | Strongly disagree | Neutral |
| Strongly disagree | Disagree | Strongly disagree | Neutral |
| Disagree | Disagree | Strongly disagree | Disagree |
| Strongly disagree | Neutral | Strongly disagree | Strongly agree |
| Neutral | Neutral | Strongly disagree | Strongly agree |
| Disagree | Disagree | Disagree | Neutral |
| Disagree | Disagree | Disagree | Agree |
| Agree | Disagree | Neutral | Agree |
| Disagree | Neutral | Disagree | Strongly agree |
| Strongly agree | Strongly agree | Strongly agree | Strongly agree |
| Strongly disagree | Strongly disagree | Strongly agree | Strongly agree |
| Disagree | Neutral | Agree | Neutral |
| Strongly disagree | Strongly disagree | Strongly disagree | Strongly agree |
| Disagree | Agree | Strongly agree | Strongly agree |

| | | | |
|---|---|---|---|
| Neutral | Agree | Strongly disagree | Neutral |
| Strongly disagree | Disagree | Disagree | Agree |
| Neutral | Neutral | Agree | Strongly agree |
| Agree | Agree | Neutral | Disagree |
| Neutral | Neutral | Neutral | Neutral |
| Disagree | Neutral | Disagree | Strongly agree |
| Disagree | Disagree | Agree | Neutral |
| Strongly agree | Strongly agree | Strongly agree | Strongly agree |
| Neutral | Strongly agree | Neutral | Agree |
| Strongly agree | Strongly agree | Neutral | Strongly disagree |
| Disagree | Strongly disagree | Strongly disagree | Agree |
| Strongly agree | Neutral | Neutral | Strongly agree |
| Strongly agree | Agree | Agree | Agree |
| Strongly agree | Agree | Strongly agree | Agree |
| Disagree | Neutral | Disagree | Strongly agree |
| Disagree | Neutral | Agree | Agree |
| Disagree | Neutral | Disagree | Neutral |
| Strongly disagree | Strongly disagree | Strongly disagree | Strongly agree |
| Strongly disagree | Agree | Strongly disagree | Strongly agree |
| Strongly agree | Strongly agree | Strongly agree | Strongly agree |
| Agree | Agree | Agree | Agree |
| Neutral | Neutral | Neutral | Strongly agree |
| Strongly disagree | Strongly disagree | Strongly disagree | Strongly agree |

| V14- I would welcome t | V15- Shared learning w | V16- Shared learning b | V17- The function of nu |
|---|---|---|---|
| Neutral | Neutral | Neutral | Agree |
| Neutral | Agree | Agree | Disagree |
| Neutral | Neutral | Agree | Disagree |
| Strongly disagree | Strongly disagree | Strongly agree | Disagree |
| Neutral | Agree | Strongly agree | Strongly agree |
| Strongly agree | Strongly agree | Strongly agree | Strongly agree |
| Agree | Agree | Strongly agree | Agree |
| Neutral | Neutral | Neutral | Neutral |
| Agree | Strongly disagree | Agree | Disagree |
| Agree | Strongly agree | Strongly agree | Disagree |
| Neutral | Strongly agree | Strongly agree | Disagree |
| Neutral | Agree | Strongly agree | Agree |
| Strongly agree | Strongly agree | Strongly agree | Disagree |
| Disagree | Neutral | Disagree | Disagree |
| Neutral | Neutral | Neutral | Neutral |
| Strongly agree | Strongly agree | Strongly agree | Disagree |
| Agree | Strongly disagree | Disagree | Strongly disagree |
| Strongly agree | Strongly agree | Strongly agree | Strongly agree |
| Agree | Agree | Agree | Neutral |
| Agree | Strongly agree | Neutral | Neutral |
| Strongly disagree | Strongly disagree | Strongly disagree | Strongly disagree |
| Agree | Agree | Agree | Agree |
| Neutral | Agree | Neutral | Neutral |
| Strongly agree | Strongly agree | Strongly agree | Strongly agree |
| Disagree | Neutral | Agree | Agree |
| Strongly agree | Strongly agree | Strongly agree | Strongly agree |
| Agree | Agree | Agree | Strongly agree |
| Agree | Agree | Agree | Disagree |
| Strongly disagree | Strongly disagree | Strongly disagree | Strongly disagree |
| Strongly disagree | Strongly disagree | Strongly disagree | Strongly disagree |
| Strongly agree | Strongly agree | Strongly agree | Disagree |
| Strongly agree | Agree | Strongly agree | Disagree |
| Agree | Agree | Agree | Agree |
| Agree | Agree | Agree | Neutral |
| Neutral | Strongly agree | Strongly agree | Strongly agree |
| Strongly agree | Strongly agree | Strongly agree | Disagree |
| Agree | Neutral | Agree | Disagree |
| Strongly agree | Strongly agree | Strongly agree | Strongly agree |
| Strongly agree | Strongly agree | Strongly agree | Strongly disagree |
| Strongly agree | Agree | Agree | Strongly disagree |
| Agree | Agree | Agree | Agree |
| Agree | Neutral | Agree | Agree |
| Agree | Agree | Strongly agree | Neutral |
| Strongly agree | Strongly agree | Strongly agree | Strongly disagree |
| Neutral | Agree | Strongly agree | Agree |
| Neutral | Neutral | Neutral | Neutral |
| Strongly agree | Agree | Agree | Disagree |
| Strongly agree | Neutral | Strongly agree | Neutral |
| Neutral | Agree | Strongly agree | Neutral |
| Neutral | Agree | Neutral | Agree |
| Strongly agree | Strongly agree | Neutral | Strongly disagree |
| Agree | Agree | Strongly agree | Disagree |

| | | | |
|---|---|---|---|
| Agree | Strongly agree | Agree | Agree |
| Agree | Agree | Agree | Agree |
| Strongly agree | Agree | Agree | Neutral |
| Strongly disagree | Agree | Agree | Agree |
| Agree | Neutral | Strongly agree | Neutral |
| Neutral | Neutral | Neutral | Neutral |
| Neutral | Neutral | Neutral | Neutral |
| Neutral | Neutral | Neutral | Neutral |
| Agree | Agree | Neutral | Agree |
| Strongly agree | Strongly agree | Strongly agree | Neutral |
| Disagree | Disagree | Disagree | Disagree |
| Strongly agree | Agree | Agree | Strongly agree |
| Strongly disagree | Strongly agree | Agree | Strongly disagree |
| Disagree | Neutral | Disagree | Neutral |
| Agree | Agree | Agree | Neutral |
| Agree | Agree | Neutral | Strongly disagree |
| Strongly agree | Agree | Agree | Neutral |
| Neutral | Agree | Neutral | Neutral |
| Neutral | Agree | Strongly agree | Neutral |
| Neutral | Neutral | Strongly agree | Strongly disagree |
| Strongly agree | Disagree | Agree | Neutral |
| Agree | Agree | Agree | Strongly disagree |
| Agree | Agree | Agree | Agree |
| Strongly agree | Strongly agree | Strongly agree | Strongly agree |
| Agree | Neutral | Neutral | Agree |
| Strongly agree | Strongly agree | Strongly agree | Strongly disagree |
| Agree | Agree | Disagree | Disagree |
| Strongly agree | Strongly agree | Strongly agree | Neutral |
| Strongly agree | Strongly agree | Neutral | Strongly agree |
| Disagree | Neutral | Agree | Agree |
| Neutral | Agree | Neutral | Strongly disagree |
| Strongly agree | Strongly agree | Strongly agree | Strongly disagree |
| Neutral | Agree | Agree | Disagree |
| Agree | Agree | Agree | Agree |
| Agree | Agree | Agree | Agree |
| Strongly disagree | Disagree | Strongly disagree | Disagree |
| Agree | Agree | Agree | Strongly agree |
| Strongly agree | Agree | Disagree | Agree |
| Agree | Agree | Neutral | Neutral |
| Agree | Agree | Agree | Agree |
| Agree | Agree | Neutral | Agree |
| Agree | Agree | Agree | Strongly agree |
| Agree | Agree | Strongly agree | Neutral |
| Agree | Agree | Agree | Agree |
| Agree | Agree | Agree | Neutral |
| Strongly agree | Strongly agree | Strongly agree | Disagree |
| Neutral | Agree | Strongly agree | Neutral |
| Agree | Agree | Agree | Strongly disagree |
| Agree | Strongly agree | Neutral | Neutral |
| Neutral | Neutral | Neutral | Neutral |
| Agree | Agree | Neutral | Agree |
| Agree | Agree | Strongly agree | Neutral |
| Agree | Agree | Agree | Strongly agree |
| Strongly agree | Strongly agree | Strongly agree | Strongly agree |
| Strongly agree | Agree | Agree | Strongly agree |
| Strongly agree | Neutral | Strongly agree | Strongly agree |
| Neutral | Agree | Agree | Agree |
| Neutral | Neutral | Neutral | Neutral |
| Strongly agree | Agree | Agree | Agree |

| | | | |
|---|---|---|---|
| Neutral | Neutral | Neutral | Neutral |
| Agree | Neutral | Strongly agree | Strongly agree |
| Neutral | Agree | Neutral | Disagree |
| Agree | Agree | Agree | Neutral |
| Strongly agree | Strongly agree | Strongly agree | Strongly agree |
| Neutral | Neutral | Agree | Agree |
| Strongly agree | Strongly agree | Strongly agree | Strongly agree |
| Neutral | Neutral | Neutral | Neutral |
| Strongly disagree | Strongly disagree | Strongly disagree | Strongly disagree |
| Agree | Agree | Agree | Agree |
| Agree | Agree | Agree | Agree |
| Strongly agree | Strongly agree | Strongly agree | Strongly agree |
| Neutral | Neutral | Agree | Strongly agree |
| Strongly agree | Strongly agree | Strongly agree | Strongly agree |
| Strongly agree | Strongly agree | Strongly agree | Strongly agree |
| Agree | Agree | Agree | Agree |
| Neutral | Neutral | Agree | Agree |
| Strongly agree | Strongly agree | Strongly agree | Strongly agree |
| Agree | Agree | Agree | Disagree |
| Strongly agree | Strongly agree | Strongly agree | Agree |
| Strongly agree | Strongly agree | Strongly agree | Disagree |
| Neutral | Agree | Neutral | Neutral |
| Agree | Strongly agree | Strongly agree | Strongly disagree |
| Neutral | Agree | Agree | Neutral |
| Neutral | Neutral | Neutral | Neutral |
| Strongly agree | Agree | Agree | Agree |
| Strongly agree | Strongly agree | Strongly agree | Neutral |
| Neutral | Strongly agree | Strongly agree | Strongly disagree |
| Strongly agree | Strongly agree | Strongly agree | Disagree |
| Agree | Strongly agree | Agree | Neutral |
| Disagree | Agree | Agree | Strongly agree |
| Disagree | Agree | Agree | Strongly agree |
| Agree | Agree | Agree | Neutral |
| Strongly agree | Strongly agree | Agree | Neutral |
| Agree | Agree | Agree | Agree |
| Strongly disagree | Agree | Agree | Strongly disagree |
| Neutral | Agree | Neutral | Neutral |
| Strongly agree | Strongly agree | Strongly agree | Strongly agree |
| Disagree | Strongly disagree | Strongly disagree | Strongly disagree |
| Disagree | Disagree | Disagree | Strongly disagree |
| Agree | Agree | Agree | Disagree |
| Neutral | Agree | Agree | Agree |
| Agree | Agree | Neutral | Strongly disagree |
| Neutral | Agree | Neutral | Agree |
| Strongly agree | Strongly agree | Strongly agree | Strongly agree |
| Strongly agree | Strongly agree | Strongly agree | Strongly disagree |
| Disagree | Strongly disagree | Strongly agree | Agree |
| Disagree | Strongly disagree | Strongly disagree | Disagree |
| Agree | Strongly agree | Agree | Disagree |
| Strongly agree | Strongly agree | Strongly agree | Strongly agree |
| Strongly agree | Strongly agree | Strongly agree | Strongly agree |
| Neutral | Neutral | Neutral | Agree |
| Strongly agree | Strongly agree | Strongly agree | Strongly agree |
| Agree | Agree | Agree | Disagree |
| Disagree | Agree | Disagree | Strongly disagree |
| Agree | Agree | Agree | Agree |
| Strongly agree | Strongly agree | Strongly agree | Neutral |
| Strongly agree | Strongly agree | Neutral | Neutral |
| Strongly agree | Strongly agree | Strongly agree | Strongly disagree |

| | | | |
|---|---|---|---|
| Agree | Neutral | Neutral | Strongly agree |
| Disagree | Disagree | Strongly disagree | Strongly disagree |
| Disagree | Neutral | Agree | Disagree |
| Agree | Strongly agree | Agree | Strongly disagree |
| Agree | Agree | Agree | Neutral |
| Neutral | Agree | Agree | Disagree |
| Agree | Strongly agree | Strongly agree | Disagree |
| Agree | Agree | Agree | Disagree |
| Strongly agree | Strongly agree | Strongly agree | Strongly disagree |
| Strongly agree | Strongly agree | Agree | Agree |
| Strongly disagree | Strongly disagree | Strongly disagree | Strongly disagree |
| Agree | Agree | Agree | Agree |
| Strongly disagree | Strongly disagree | Strongly disagree | Strongly disagree |
| Agree | Agree | Agree | Agree |
| Strongly agree | Strongly agree | Strongly agree | Strongly agree |
| Agree | Agree | Agree | Neutral |
| Agree | Agree | Strongly agree | Agree |
| Strongly agree | Strongly agree | Strongly agree | Strongly agree |
| Agree | Agree | Agree | Disagree |
| Neutral | Agree | Neutral | Disagree |
| Strongly agree | Strongly agree | Strongly agree | Disagree |
| Agree | Agree | Agree | Neutral |
| Strongly agree | Strongly agree | Strongly agree | Strongly agree |
| Strongly agree | Strongly agree | Strongly agree | Neutral |
| Agree | Agree | Agree | Neutral |
| Strongly agree | Strongly agree | Strongly agree | Agree |
| Agree | Neutral | Agree | Strongly disagree |
| Strongly agree | Strongly agree | Strongly agree | Strongly disagree |
| Strongly agree | Strongly agree | Strongly agree | Strongly agree |
| Agree | Agree | Agree | Strongly agree |
| Agree | Agree | Agree | Neutral |
| Agree | Agree | Agree | Neutral |
| Agree | Neutral | Neutral | Agree |
| Agree | Agree | Disagree | Disagree |
| Strongly agree | Strongly agree | Strongly agree | Disagree |
| Strongly agree | Neutral | Strongly agree | Agree |
| Agree | Agree | Agree | Agree |
| Agree | Neutral | Agree | Agree |
| Strongly agree | Strongly agree | Strongly agree | Strongly disagree |
| Agree | Disagree | Strongly disagree | Agree |
| Strongly disagree | Strongly disagree | Strongly disagree | Strongly disagree |
| Agree | Agree | Agree | Disagree |
| Strongly agree | Strongly agree | Strongly agree | Strongly agree |
| Agree | Agree | Agree | Strongly agree |
| Agree | Strongly agree | Disagree | Strongly disagree |
| Strongly disagree | Strongly disagree | Strongly disagree | Strongly disagree |
| Agree | Neutral | Neutral | Neutral |
| Strongly agree | Agree | Strongly agree | Agree |
| Strongly agree | Strongly agree | Strongly agree | Strongly agree |
| Strongly agree | Strongly agree | Strongly agree | Strongly agree |
| Strongly agree | Strongly agree | Neutral | Strongly agree |
| Strongly agree | Strongly agree | Strongly agree | Agree |
| Strongly agree | Strongly agree | Strongly agree | Strongly disagree |
| Strongly agree | Strongly agree | Strongly agree | Strongly agree |
| Strongly agree | Strongly agree | Strongly agree | Strongly disagree |
| Strongly disagree | Agree | Agree | Agree |
| Strongly agree | Agree | Strongly agree | Strongly disagree |
| Neutral | Neutral | Neutral | Neutral |
| Disagree | Agree | Disagree | Disagree |

| | | | |
|---|---|---|---|
| Strongly agree | Agree | Agree | Agree |
| Agree | Neutral | Neutral | Agree |
| Strongly agree | Strongly agree | Strongly agree | Strongly disagree |
| Disagree | Agree | Neutral | Strongly disagree |
| Strongly agree | Strongly agree | Strongly agree | Disagree |
| Agree | Agree | Strongly agree | Neutral |
| Agree | Agree | Neutral | Disagree |
| Agree | Strongly agree | Strongly agree | Strongly disagree |
| Agree | Agree | Agree | Disagree |
| Agree | Strongly agree | Strongly agree | Agree |
| Agree | Agree | Agree | Neutral |
| Neutral | Agree | Agree | Strongly disagree |
| Agree | Strongly agree | Strongly agree | Strongly disagree |
| Agree | Strongly agree | Strongly agree | Disagree |
| Agree | Agree | Agree | Agree |
| Strongly agree | Strongly agree | Strongly agree | Agree |
| Neutral | Agree | Agree | Disagree |
| Strongly agree | Agree | Strongly agree | Disagree |
| Strongly agree | Strongly agree | Strongly agree | Strongly agree |
| Agree | Agree | Strongly agree | Disagree |
| Strongly agree | Strongly agree | Strongly agree | Strongly disagree |
| Agree | Agree | Agree | Strongly disagree |
| Agree | Agree | Agree | Neutral |
| Strongly disagree | Disagree | Strongly disagree | Strongly disagree |
| Neutral | Agree | Agree | Agree |
| Strongly agree | Strongly agree | Strongly agree | Strongly disagree |
| Agree | Agree | Strongly agree | Strongly disagree |
| Strongly agree | Strongly agree | Strongly agree | Strongly agree |
| Neutral | Strongly agree | Strongly agree | Strongly disagree |
| Strongly agree | Strongly agree | Agree | Strongly agree |
| Strongly agree | Strongly agree | Strongly agree | Neutral |
| Neutral | Neutral | Strongly disagree | Disagree |
| Agree | Strongly agree | Strongly agree | Strongly disagree |
| Agree | Agree | Agree | Agree |
| Neutral | Agree | Neutral | Agree |
| Agree | Agree | Neutral | Neutral |
| Neutral | Neutral | Neutral | Neutral |
| Neutral | Strongly agree | Agree | Strongly agree |
| Agree | Agree | Agree | Agree |
| Agree | Agree | Strongly agree | Disagree |
| Strongly agree | Agree | Strongly agree | Strongly agree |
| Neutral | Neutral | Neutral | Neutral |
| Neutral | Agree | Agree | Agree |
| Strongly agree | Strongly agree | Strongly agree | Strongly disagree |
| Agree | Agree | Agree | Neutral |
| Disagree | Strongly disagree | Strongly agree | Agree |
| Strongly disagree | Strongly disagree | Neutral | Disagree |
| Neutral | Agree | Neutral | Disagree |
| Agree | Strongly agree | Strongly agree | Strongly disagree |
| Neutral | Strongly agree | Strongly agree | Strongly agree |
| Neutral | Neutral | Neutral | Neutral |
| Agree | Agree | Agree | Disagree |
| Neutral | Neutral | Strongly agree | Strongly agree |
| Neutral | Strongly agree | Strongly agree | Strongly disagree |
| Strongly agree | Strongly agree | Strongly agree | Strongly agree |
| Strongly agree | Strongly agree | Strongly agree | Strongly disagree |
| Disagree | Neutral | Neutral | Agree |
| Strongly agree | Strongly agree | Strongly agree | Strongly disagree |
| Neutral | Agree | Agree | Agree |

| | | | |
|---|---|---|---|
| Agree | Agree | Neutral | Strongly disagree |
| Agree | Disagree | Agree | Strongly disagree |
| Agree | Strongly agree | Agree | Strongly agree |
| Disagree | Neutral | Agree | Neutral |
| Neutral | Agree | Neutral | Neutral |
| Agree | Strongly agree | Agree | Strongly agree |
| Strongly agree | Neutral | Neutral | Agree |
| Strongly agree | Strongly agree | Strongly agree | Strongly agree |
| Neutral | Agree | Agree | Strongly agree |
| Neutral | Strongly disagree | Strongly disagree | Neutral |
| Agree | Agree | Strongly agree | Neutral |
| Agree | Agree | Agree | Agree |
| Agree | Agree | Agree | Agree |
| Agree | Agree | Strongly agree | Neutral |
| Strongly agree | Strongly agree | Strongly agree | Strongly disagree |
| Agree | Neutral | Neutral | Strongly disagree |
| Strongly agree | Strongly agree | Strongly agree | Disagree |
| Strongly agree | Strongly agree | Strongly agree | Strongly agree |
| Agree | Disagree | Neutral | Agree |
| Strongly agree | Strongly agree | Strongly agree | Strongly agree |
| Agree | Agree | Agree | Agree |
| Strongly agree | Strongly agree | Strongly agree | Strongly disagree |
| Strongly agree | Strongly agree | Strongly agree | Strongly agree |

| V18- I'm not sure what | V19- I have to acquire much more knowledge and skills than other hea |
|---|---|
| Agree | Agree |
| Disagree | Agree |
| Disagree | Disagree |
| Strongly agree | Disagree |
| Strongly agree | Strongly agree |
| Disagree | Strongly agree |
| Disagree | Agree |
| Neutral | Neutral |
| Disagree | Agree |
| Strongly disagree | Neutral |
| Neutral | Agree |
| Disagree | Strongly agree |
| Strongly disagree | Neutral |
| Strongly agree | Strongly agree |
| Neutral | Neutral |
| Disagree | Neutral |
| Strongly disagree | Strongly agree |
| Disagree | Strongly agree |
| Neutral | Agree |
| Neutral | Agree |
| Strongly disagree | Strongly disagree |
| Disagree | Strongly agree |
| Disagree | Neutral |
| Neutral | Strongly agree |
| Disagree | Neutral |
| Disagree | Strongly agree |
| Strongly agree | Strongly agree |
| Disagree | Agree |
| Strongly disagree | Strongly disagree |
| Disagree | Strongly agree |
| Agree | Strongly agree |
| Disagree | Strongly agree |
| Strongly disagree | Agree |
| Disagree | Strongly agree |
| Neutral | Strongly agree |
| Disagree | Neutral |
| Disagree | Neutral |
| Strongly agree | Neutral |
| Disagree | Disagree |
| Disagree | Neutral |
| Neutral | Neutral |
| Agree | Neutral |
| Strongly agree | Strongly agree |
| Strongly disagree | Agree |
| Agree | Agree |
| Neutral | Neutral |
| Disagree | Neutral |
| Strongly disagree | Agree |
| Neutral | Agree |
| Neutral | Disagree |
| Strongly disagree | Disagree |
| Strongly disagree | Strongly agree |

| | |
|---|---|
| Neutral | Neutral |
| Strongly disagree | Strongly agree |
| Strongly disagree | Agree |
| Agree | Disagree |
| Strongly disagree | Neutral |
| Disagree | Agree |
| Neutral | Neutral |
| Neutral | Neutral |
| Neutral | Strongly agree |
| Strongly disagree | Agree |
| Disagree | Strongly disagree |
| Disagree | Agree |
| Disagree | Agree |
| Disagree | Disagree |
| Agree | Strongly agree |
| Disagree | Disagree |
| Agree | Strongly agree |
| Neutral | Neutral |
| Neutral | Neutral |
| Disagree | Neutral |
| Disagree | Agree |
| Strongly disagree | Strongly disagree |
| Strongly disagree | Agree |
| Strongly agree | Strongly agree |
| Agree | Neutral |
| Strongly disagree | Strongly agree |
| Disagree | Neutral |
| Disagree | Neutral |
| Strongly disagree | Agree |
| Disagree | Disagree |
| Agree | Strongly agree |
| Disagree | Neutral |
| Disagree | Strongly agree |
| Agree | Agree |
| Agree | Agree |
| Strongly disagree | Strongly disagree |
| Neutral | Agree |
| Disagree | Strongly agree |
| Neutral | Agree |
| Agree | Agree |
| Strongly agree | Strongly agree |
| Strongly disagree | Neutral |
| Neutral | Agree |
| Disagree | Agree |
| Disagree | Agree |
| Disagree | Strongly agree |
| Neutral | Strongly agree |
| Disagree | Neutral |
| Agree | Disagree |
| Neutral | Neutral |
| Agree | Disagree |
| Neutral | Agree |
| Strongly agree | Strongly agree |
| Strongly agree | Strongly agree |
| Disagree | Neutral |
| Disagree | Strongly agree |
| Strongly disagree | Agree |
| Neutral | Neutral |
| Disagree | Neutral |

| | |
|---|---|
| Disagree | Disagree |
| Strongly disagree | Strongly agree |
| Disagree | Neutral |
| Strongly disagree | Neutral |
| Disagree | Strongly agree |
| Neutral | Neutral |
| Strongly agree | Strongly agree |
| Agree | Agree |
| Strongly disagree | Strongly disagree |
| Agree | Agree |
| Agree | Agree |
| Strongly agree | Strongly agree |
| Disagree | Disagree |
| Strongly agree | Strongly agree |
| Strongly agree | Strongly agree |
| Agree | Agree |
| Disagree | Agree |
| Strongly agree | Strongly agree |
| Neutral | Neutral |
| Disagree | Strongly agree |
| Disagree | Disagree |
| Neutral | Agree |
| Disagree | Neutral |
| Neutral | Agree |
| Neutral | Neutral |
| Agree | Agree |
| Agree | Neutral |
| Strongly agree | Neutral |
| Neutral | Strongly agree |
| Disagree | Strongly agree |
| Neutral | Agree |
| Agree | Agree |
| Strongly agree | Strongly agree |
| Agree | Agree |
| Agree | Neutral |
| Disagree | Agree |
| Neutral | Neutral |
| Strongly agree | Strongly agree |
| Neutral | Strongly disagree |
| Strongly disagree | Disagree |
| Neutral | Neutral |
| Agree | Agree |
| Disagree | Disagree |
| Neutral | Agree |
| Strongly agree | Strongly agree |
| Strongly disagree | Neutral |
| Disagree | Neutral |
| Neutral | Disagree |
| Strongly disagree | Agree |
| Neutral | Agree |
| Strongly agree | Strongly agree |
| Agree | Neutral |
| Strongly agree | Strongly agree |
| Agree | Neutral |
| Disagree | Disagree |
| Agree | Agree |
| Strongly disagree | Agree |
| Strongly disagree | Strongly agree |
| Strongly disagree | Strongly agree |

| | |
|---|---|
| Neutral | Strongly agree |
| Disagree | Strongly disagree |
| Neutral | Strongly disagree |
| Disagree | Neutral |
| Neutral | Neutral |
| Disagree | Agree |
| Disagree | Neutral |
| Disagree | Neutral |
| Strongly disagree | Neutral |
| Disagree | Neutral |
| Strongly disagree | Strongly disagree |
| Agree | Agree |
| Strongly disagree | Strongly disagree |
| Agree | Agree |
| Strongly agree | Strongly agree |
| Neutral | Agree |
| Agree | Agree |
| Strongly agree | Strongly agree |
| Disagree | Agree |
| Disagree | Agree |
| Disagree | Agree |
| Disagree | Neutral |
| Strongly agree | Strongly agree |
| Strongly disagree | Disagree |
| Disagree | Neutral |
| Strongly disagree | Strongly agree |
| Strongly disagree | Disagree |
| Strongly disagree | Strongly disagree |
| Strongly agree | Strongly agree |
| Strongly disagree | Neutral |
| Disagree | Disagree |
| Neutral | Agree |
| Neutral | Agree |
| Strongly disagree | Agree |
| Neutral | Neutral |
| Neutral | Neutral |
| Agree | Agree |
| Agree | Agree |
| Strongly disagree | Strongly agree |
| Disagree | Neutral |
| Strongly disagree | Disagree |
| Disagree | Neutral |
| Disagree | Agree |
| Disagree | Agree |
| Strongly disagree | Disagree |
| Strongly disagree | Strongly disagree |
| Strongly agree | Strongly agree |
| Neutral | Strongly agree |
| Neutral | Agree |
| Strongly agree | Strongly agree |
| Neutral | Strongly agree |
| Strongly disagree | Neutral |
| Strongly disagree | Disagree |
| Strongly agree | Neutral |
| Strongly disagree | Agree |
| Neutral | Neutral |
| Agree | Neutral |
| Neutral | Neutral |
| Disagree | Strongly agree |

| | |
|---|---|
| Agree | Strongly agree |
| Neutral | Strongly agree |
| Strongly disagree | Strongly agree |
| Strongly disagree | Strongly agree |
| Neutral | Strongly agree |
| Disagree | Agree |
| Strongly disagree | Agree |
| Neutral | Strongly agree |
| Strongly disagree | Disagree |
| Strongly disagree | Neutral |
| Neutral | Neutral |
| Strongly disagree | Disagree |
| Neutral | Neutral |
| Neutral | Disagree |
| Agree | Agree |
| Disagree | Agree |
| Disagree | Agree |
| Neutral | Agree |
| Neutral | Neutral |
| Disagree | Neutral |
| Disagree | Strongly agree |
| Agree | Strongly agree |
| Agree | Neutral |
| Disagree | Strongly disagree |
| Agree | Agree |
| Strongly disagree | Strongly agree |
| Neutral | Agree |
| Disagree | Agree |
| Disagree | Neutral |
| Neutral | Strongly agree |
| Strongly disagree | Agree |
| Disagree | Disagree |
| Strongly disagree | Neutral |
| Agree | Agree |
| Agree | Strongly agree |
| Agree | Agree |
| Neutral | Neutral |
| Agree | Strongly agree |
| Neutral | Neutral |
| Disagree | Neutral |
| Disagree | Neutral |
| Neutral | Neutral |
| Agree | Neutral |
| Strongly disagree | Strongly agree |
| Neutral | Agree |
| Agree | Agree |
| Disagree | Strongly disagree |
| Strongly disagree | Agree |
| Strongly agree | Strongly agree |
| Disagree | Strongly agree |
| Neutral | Neutral |
| Disagree | Strongly agree |
| Disagree | Neutral |
| Agree | Neutral |
| Strongly agree | Strongly agree |
| Neutral | Strongly agree |
| Agree | Agree |
| Strongly disagree | Strongly agree |
| Neutral | Neutral |

| | |
|---|---|
| Strongly disagree | Neutral |
| Agree | Agree |
| Neutral | Strongly agree |
| Neutral | Neutral |
| Disagree | Agree |
| Neutral | Strongly agree |
| Strongly disagree | Agree |
| Strongly agree | Agree |
| Disagree | Strongly agree |
| Agree | Strongly disagree |
| Agree | Strongly agree |
| Agree | Agree |
| Agree | Agree |
| Disagree | Strongly agree |
| Strongly disagree | Neutral |
| Strongly disagree | Agree |
| Neutral | Neutral |
| Disagree | Strongly agree |
| Strongly agree | Strongly agree |
| Strongly agree | Strongly agree |
| Agree | Agree |
| Strongly disagree | Strongly agree |
| Strongly agree | Neutral |

lth-care students
