# Supplementary material for: Exploring perception and attitude of nursing students towards interprofessional education in Saudi Arabia
Source: PLoS One. 2024 Oct 4;19(10):e0311570. doi: 10.1371/journal.pone.0311570 (PMC11452034; doi:10.1371/journal.pone.0311570)
Supplement: Attachment — Submitted filename: Exploring Perception and Attitude of Nursing Student.pdf [file pone.0311570.s005.pdf]

**Exploring Perception and Attitude of Nursing Students Towards Interprofessional Education in Saudi Arabia**

**Generally**:

- Idea of the research is good in an exciting development of the field.
- The paper is generally well written and structured.
- The study is understandable. The idea of the research is good in an exciting development of the field and an addition to the existing literature. This is an interesting study.
- No critical information missing.
- The study article is acceptable after minor revisions as per the reviewer's comments. Below I have provided few remarks:

**Abstract:**

The abstract provides a clear and concise summary of the study, including the aim, methodology, key findings, and conclusion. It effectively sets the stage for the reader to understand the scope and significance of the research.

**Introduction**:

The introduction is well-structured, providing a comprehensive background on Interprofessional Education (IPE) and its importance in healthcare. The authors effectively highlight the gap in research regarding nursing students' attitudes towards high-fidelity simulation (HFS) IPE in Saudi Arabia.

**Methods**

The methodology section is detailed and transparent. The use of a descriptive cross-sectional design and convenience sampling is appropriate for the study's objectives. However, the reliance on convenience sampling may introduce bias, which is acknowledged by the authors.

**Results**

The results are presented clearly, with appropriate use of tables to summarize the data. The statistical analysis is thorough and well-explained.

**Discussion:**

The discussion section effectively interprets the results in the context of existing literature. The authors provide a balanced view of the benefits and challenges of IPE, and they discuss the implications of their findings for nursing education in Saudi Arabia.

**Conclusion**

The conclusion succinctly summarizes the key findings and their implications. The authors emphasize the potential benefits of integrating HFS into IPE and suggest areas for future research.

**References**

The references in the document are comprehensive and relevant to the study on Interprofessional Education (IPE) among nursing students. Here are some key points about the references:

- Diversity of Sources
- Recent Studies
- Relevant Topics
- Credible Journals

**Strengths:**

- Comprehensive background and literature review.
- Clear and detailed methodology.
- Thorough statistical analysis.
- Balanced discussion of results.

The research does have some **limitations**:

- Sample Limitation: The study only involved nursing students from one university, which limits the generalizability of the findings to other institutions or regions.
- Cross-Sectional Design: The research used a descriptive and cross-sectional approach, which precludes tracking changes over time and understanding long-term impacts.
- Convenience Sampling: The sampling was done by convenience and was not probabilistic, which might introduce bias and affect the representativeness of the sample.
- Exclusion of Early-Year Students: First- and second-year students were excluded from the study, which means the findings do not reflect the attitudes and readiness of all undergraduate nursing students.
- **These limitations suggest that while the findings are valuable, they should be interpreted with caution and further research is needed to confirm and expand upon these results.**

Overall, this study makes a valuable contribution to the understanding of nursing students' attitudes towards IPE in Saudi Arabia and provides a solid foundation for future research in this area.
