# Supplementary material for: Exploring perception and attitude of nursing students towards interprofessional education in Saudi Arabia
Source: PLoS One. 2024 Oct 4;19(10):e0311570. doi: 10.1371/journal.pone.0311570 (PMC11452034; doi:10.1371/journal.pone.0311570)
Supplement: Attachment — Submitted filename: PLOSE ONE F.docx [file pone.0311570.s003.docx]

Dear Authors,

Thanks for choosing PLOS ONE. I hope my feedback is helpful for you.

Abstract: please add the sampling method (i.e., convenience sampling method).

Methods: Please address the reporting guidelines (i.e., STROBE). For more information, please check the link below <https://www.equator-network.org>

Elaborate more on the data collection procedure

The reference for the RIPLS was not addressed within the methods section. Please cite the original study and report the reliability and validity of the instrument.

Please replace this statement: " Statistical significance was determined by p ≤ .05." to " statistical significance was determined by *p* <.05.

I recommend you to modify the heading of Table 4 to "The association between demographics' characteristics and RIPLS"

The limitations of the study were too brief. Please address the major limitations associated with the methodology (i.e., sampling method, research design, etc...) and how these limitations affect the generalizability of the findings.

Address the implications for future research and practice in a separate heading.
