# Supplementary material for: Exploring perception and attitude of nursing students towards interprofessional education in Saudi Arabia
Source: PLoS One. 2024 Oct 4;19(10):e0311570. doi: 10.1371/journal.pone.0311570 (PMC11452034; doi:10.1371/journal.pone.0311570)
Supplement: Attachment — Submitted filename: Response to reviewers 2.docx [file pone.0311570.s004.docx]

Reviewers' comments:

Reviewer's Responses to Questions

**Comments to the Author**

1. Is the manuscript technically sound, and do the data support the conclusions?

The manuscript must describe a technically sound piece of scientific research with data that supports the conclusions. Experiments must have been conducted rigorously, with appropriate controls, replication, and sample sizes. The conclusions must be drawn appropriately based on the data presented.

Reviewer #1: Yes

Reviewer #2: No

2. Has the statistical analysis been performed appropriately and rigorously?

Reviewer #1: I Don't Know

Reviewer #2: No

3. Have the authors made all data underlying the findings in their manuscript fully available?

The [PLOS Data policy](https://urldefense.com/v3/__http:/www.plosone.org/static/policies.action*sharing__;Iw!!DoQ1b8aHUAUitbY2jRg!lqRr3a42hcC-p4ZrBFAygu2-cWELwWZq0-SYyk6frjwVuIZ2TE4TD51CUtjuUl1zW8xZ-X2zhk4sQvhhnqB3QA$) requires authors to make all data underlying the findings described in their manuscript fully available without restriction, with rare exception (please refer to the Data Availability Statement in the manuscript PDF file). The data should be provided as part of the manuscript or its supporting information, or deposited to a public repository. For example, in addition to summary statistics, the data points behind means, medians and variance measures should be available. If there are restrictions on publicly sharing data—e.g. participant privacy or use of data from a third party—those must be specified.

Reviewer #1: No

Reviewer #2: Yes

4. Is the manuscript presented in an intelligible fashion and written in standard English?

PLOS ONE does not copyedit accepted manuscripts, so the language in submitted articles must be clear, correct, and unambiguous. Any typographical or grammatical errors should be corrected at revision, so please note any specific errors here.

Reviewer #1: Yes

Reviewer #2: Yes

5. Review Comments to the Author

Please use the space provided to explain your answers to the questions above. You may also include additional comments for the author, including concerns about dual publication, research ethics, or publication ethics. (Please upload your review as an attachment if it exceeds 20,000 characters)

**Reviewer #1:** I would like to, firstly, congratulate the authors for their effort and dedication in writing this scientific article. I hope that the following considerations will further strengthen your work.

In the introduction, it would be pertinent to include the current state-of-the-art regarding nursing students' readiness and attitudes for IPE. We have existing studies that have analyzed the attitude and readiness of nursing and healthcare students in simulation scenarios. Therefore, I suggest addressing existing studies in the field, the uniqueness of your study, and/or the relevance of your study, considering the simulation scenario. Students underwent a high-fidelity simulation cenario, and I noticed that it was not adequately explored throughout your scientific article, even though it was integrated into your aim. Integrating this aspect into both the discussion and conclusion sections would be beneficial, as it is through this pedagogical intervention that readiness and attitudes for IPE was analyzed, thus serving as a important component in the interpretation of the gathered data.

In the methodology, in the "Study design, Setting, and population" section, it would be useful to characterize/describe the university and, if possible, the nursing course. It is important in the methodology to also describe the high-fidelity simulation, as it is an important element, being the pedagogical intervention used to assess attitudes and readiness for interprofessional education and practice. In line 88, I suggest describing the name of the platform used to distribute online questionnaires to participants.

In the methodology, between lines 93 and 94, I wondered if validation occurred through the pilot study described later? If so, it's pertinent to indicate the pilot study here. For example: "A validated self-administered online questionnaire was administered following a pilot study." It is also pertinent to change the words to avoid repetition of "self-administered" and "administered."

In the discussion, in line 180, I noticed that two of these cited studies present results from faculty members and educators. It would be interesting to cite and relate to your results studies that examined attitudes using the RIPLS in nursing students during realistic simulation. For example: (This is optional and should only be taken as a suggestion for the improvement of the manuscript): 

Examining interprofessional learning perceptions among students in a simulation-based operating room team training experience. DOI: 10.1080/13561820.2018.1513464 

Does Interprofessional Scenario-Based Simulation Training Change Attitudes Towards Interprofessional Learning - A Pretest-Posttest Study. DOI: 10.2147/JMDH.S370100

Effects of High-Fidelity Simulation on Physical Therapy and Nursing Students' Attitudes Toward Interprofessional Learning and Collaboration. DOI: 10.3928/01484834-20170712-03

Effects of a single interprofessional simulation session on medical and nursing students' attitudes toward interprofessional learning and professional identity: a questionnaire study. DOI: 10.1186/s12909-020-1971-6

Implementation and evaluation of an interprofessional simulation-based education program for undergraduate nursing students in operating room nursing education: a randomized controlled trial. DOI: 10.1186/s12909-015-0400-8

In the discussion, between lines 200 and 203, we note that realistic simulation is an important aspect to consider in the results you identified regarding attitude and readiness in nursing students.

What is the reference/citation for the passage in the discussion "Recently, however, there has been a shift in emphasis toward the concept that leadership should be determined by the context in which the team operates"?

In line 226, it is appropriate to clarify in the writing that it refers to "nursing students."

In your discussion, in line 235, how does this finding "however, the academic year was significantly related to the mean total score for RIPLS (p ≤ 0.05)” relate to what we have in the scientific literature? In line 241, replace the comma with a period.

In your conclusions, it is important to include information about the high-fidelity simulation scenario, as there was an interesting connection in the discussion with the use of the simulation. This consideration also applies to the conclusion in your abstract.

**Reviewer #2:** Dear Authors,
Thanks for choosing PLOS ONE. I hope my feedback is helpful for you.
Abstract: please add the sampling method (i.e., convenience sampling method).
Methods: Please address the reporting guidelines (i.e., STROBE). For more information, please check the link below [https://www.equator-network.org](https://urldefense.com/v3/__https:/www.equator-network.org__;!!DoQ1b8aHUAUitbY2jRg!lqRr3a42hcC-p4ZrBFAygu2-cWELwWZq0-SYyk6frjwVuIZ2TE4TD51CUtjuUl1zW8xZ-X2zhk4sQvjTIZQh2w$)
Elaborate more on the data collection procedure
The reference for the RIPLS was not addressed within the methods section. Please cite the original study and report the reliability and validity of the instrument.
Please replace this statement: " Statistical significance was determined by p ≤ .05." to " statistical significance was determined by p <.05.
I recommend you to modify the heading of Table 4 to "The association between demographics' characteristics and RIPLS"
The limitations of the study were too brief. Please address the major limitations associated with the methodology (i.e., sampling method, research design, etc...) and how these limitations affect the generalizability of the findings.
Address the implications for future research and practice in a separate heading.

6. PLOS authors have the option to publish the peer review history of their article ([what does this mean?](https://urldefense.com/v3/__https:/journals.plos.org/plosone/s/editorial-and-peer-review-process*loc-peer-review-history__;Iw!!DoQ1b8aHUAUitbY2jRg!lqRr3a42hcC-p4ZrBFAygu2-cWELwWZq0-SYyk6frjwVuIZ2TE4TD51CUtjuUl1zW8xZ-X2zhk4sQvjWr_KR1w$)). If published, this will include your full peer review and any attached files.


If you choose “no”, your identity will remain anonymous but your review may still be made public.


**Do you want your identity to be public for this peer review?** For information about this choice, including consent withdrawal, please see our [Privacy Policy](https://urldefense.com/v3/__https:/www.plos.org/privacy-policy__;!!DoQ1b8aHUAUitbY2jRg!lqRr3a42hcC-p4ZrBFAygu2-cWELwWZq0-SYyk6frjwVuIZ2TE4TD51CUtjuUl1zW8xZ-X2zhk4sQvjImo3S8A$).

Reviewer #1: No

Reviewer #2: **Yes:** Dr. Adnan Innab
