# Supplementary material for: Exploring perception and attitude of nursing students towards interprofessional education in Saudi Arabia
Source: PLoS One. 2024 Oct 4;19(10):e0311570. doi: 10.1371/journal.pone.0311570 (PMC11452034; doi:10.1371/journal.pone.0311570)
Supplement: Attachment — Submitted filename: Response to reviewers.docx [file pone.0311570.s006.docx]

Reviewers' comments:

Reviewer's Responses to Questions

**Comments to the Author**

1. If the authors have adequately addressed your comments raised in a previous round of review and you feel that this manuscript is now acceptable for publication, you may indicate that here to bypass the “Comments to the Author” section, enter your conflict of interest statement in the “Confidential to Editor” section, and submit your "Accept" recommendation.

Reviewer #3: (No Response)

Reviewer #4: All comments have been addressed

2. Is the manuscript technically sound, and do the data support the conclusions?

The manuscript must describe a technically sound piece of scientific research with data that supports the conclusions. Experiments must have been conducted rigorously, with appropriate controls, replication, and sample sizes. The conclusions must be drawn appropriately based on the data presented.

Reviewer #3: Yes

Reviewer #4: Yes

3. Has the statistical analysis been performed appropriately and rigorously?

Reviewer #3: Yes

Reviewer #4: Yes

4. Have the authors made all data underlying the findings in their manuscript fully available?

The [PLOS Data policy](https://urldefense.com/v3/__http:/c05y1x9s.r.us-east-2.awstrack.me/L0/http:*2F*2Fwww.plosone.org*2Fstatic*2Fpolicies.action*23sharing/1/010f0191afb270d4-47421611-6f0e-4878-9e4e-ae460b7b5824-000000/D2lGZGz8IruMWJ4hNhREeGZGxQI=174__;JSUlJSU!!DoQ1b8aHUAUitbY2jRg!iRa78_hdbiDcNHAJjUC03uuAMaxtbRcl1uLbyxykYrSwi7qTfkLhNBAKflMGj6t772Md2hzf1U7G-16iskJTyw$) requires authors to make all data underlying the findings described in their manuscript fully available without restriction, with rare exception (please refer to the Data Availability Statement in the manuscript PDF file). The data should be provided as part of the manuscript or its supporting information, or deposited to a public repository. For example, in addition to summary statistics, the data points behind means, medians and variance measures should be available. If there are restrictions on publicly sharing data—e.g. participant privacy or use of data from a third party—those must be specified.

Reviewer #3: Yes

Reviewer #4: Yes

5. Is the manuscript presented in an intelligible fashion and written in standard English?

PLOS ONE does not copyedit accepted manuscripts, so the language in submitted articles must be clear, correct, and unambiguous. Any typographical or grammatical errors should be corrected at revision, so please note any specific errors here.

Reviewer #3: Yes

Reviewer #4: Yes

6. Review Comments to the Author

Please use the space provided to explain your answers to the questions above. You may also include additional comments for the author, including concerns about dual publication, research ethics, or publication ethics. (Please upload your review as an attachment if it exceeds 20,000 characters)

Reviewer #3: Thank you for considering me to review this manuscript. The manuscript analyses the perception and attitude of nursing students towards interprofessional education (IPE). It certainly highlights the importance of IPE in nursing education for better understanding, learning, and integration in the healthcare team. However, there are few recommendations which authors can consider to improve the manuscript.
Methods
• Study Design, Setting, and Population
The authors are suggested to justify how the sample size of 320 nursing students is sufficient for the research question in line 111. Also give information about the sample size calculation in the text.
Results
The authors framed the results well. However, following points in my opinion are recommended:
• Teamwork and Collaboration
In line 155 the authors are advised to justify how they classified the 5-point Likert scale on 2-point scale of agree and disagree values. It is suggested to discuss it in text also.
Discussion
• Study Limitations
The authors are recommended to talk about the risk of bias by using convenience sampling in the text.
• Implications for Future Research and Practice
The authors discussed almost every possible implication. However, in line 276 along with IPE, the authors are recommended to talk about the economical aspect of implementation of IPE in educating the nursing students. For better insights about the interprofessional education and collaboration, the authors may refer to the following study (This is optional and should only be taken as a suggestion for the improvement of the manuscript).
1. DOI: 10.2147/RMHP.S296113

Reviewer #4: (No Response)

7. PLOS authors have the option to publish the peer review history of their article ([what does this mean?](https://urldefense.com/v3/__https:/c05y1x9s.r.us-east-2.awstrack.me/L0/https:*2F*2Fjournals.plos.org*2Fplosone*2Fs*2Feditorial-and-peer-review-process*23loc-peer-review-history/1/010f0191afb270d4-47421611-6f0e-4878-9e4e-ae460b7b5824-000000/Nna7ukBFZhquHhbfzi3HgOKygTE=174__;JSUlJSUl!!DoQ1b8aHUAUitbY2jRg!iRa78_hdbiDcNHAJjUC03uuAMaxtbRcl1uLbyxykYrSwi7qTfkLhNBAKflMGj6t772Md2hzf1U7G-14Th4SOHA$)). If published, this will include your full peer review and any attached files.


If you choose “no”, your identity will remain anonymous but your review may still be made public.


**Do you want your identity to be public for this peer review?** For information about this choice, including consent withdrawal, please see our [Privacy Policy](https://urldefense.com/v3/__https:/c05y1x9s.r.us-east-2.awstrack.me/L0/https:*2F*2Fwww.plos.org*2Fprivacy-policy/1/010f0191afb270d4-47421611-6f0e-4878-9e4e-ae460b7b5824-000000/fm_NFul16fF8zlCahGETRCWqbEE=174__;JSUl!!DoQ1b8aHUAUitbY2jRg!iRa78_hdbiDcNHAJjUC03uuAMaxtbRcl1uLbyxykYrSwi7qTfkLhNBAKflMGj6t772Md2hzf1U7G-156MALa7w$).

Reviewer #3: No

Reviewer #4: No
